# Two Types of Liquid Phase Separation Induced by Soft Centrifugation in Aqueous Ethyl Acetate Using Ethanol as Cosolvent

**DOI:** 10.34133/research.0026

**Published:** 2023-01-16

**Authors:** Helmut Cölfen, Rose Rosenberg, Dirk Haffke, Simon Stemplinger, Thomas Zemb, Dominik Horinek

**Affiliations:** ^1^ Physical Chemistry, Department of Chemistry, University of Konstanz, Universitätsstr. 10, D-78457 Konstanz, Germany.; ^2^ Institute for Separation Chemistry ICSM U Montpellier/CEA/CNRS/ENSCM, Marcoule, France.; ^3^ Institute of Physical and Theoretical Chemistry, University of Regensburg, D-93040 Regensburg, Germany.

## Abstract

Water/ethyl acetate/ethanol is widely used as a “green” extractant system. We show that 2 different types of phase separation can be induced upon centrifugation in this ternary system using ethanol as a cosolvent of water and ethyl acetate: centrifuge-induced criticality and centrifuge-induced emulsification. The expected composition profiles of samples after centrifugation can be represented by bent lines in a ternary phase diagram when gravitational energy is added to the free energy of mixing. The experimental equilibrium composition profiles behave qualitatively as expected and can be predicted using a phenomenological theory of mixing. The concentration gradients are small except near the critical point, as expected for small molecules. Nevertheless, they are usable when accompanied by temperature cycles. These findings open new possibilities of centrifugal separation, even if control is delicate during temperature cycles. These schemes are accessible even at relatively low centrifugation speed for molecules that float and sediment with apparent molar masses several hundred times larger than the molecular mass.

## Introduction

Nearly a century after Svedberg introduced analytical ultracentrifugation (AUC) with centrifugal fields exceeding 100 000 g, analytical centrifugation remains to have a strong focus on the field of biomacromolecules [1]. However, centrifugation has a broader field of applicability. We have recently shown that centrifugation can lead to phase separation of simple binary mixtures of linear alcohols and alkanes even far from the critical point (CP) [2]. The experimental determination of the extraction efficiency in the vicinity of CPs of poorly miscible fluids has been reported, however, without predictive modeling of the chemical potential involved available [3]. Remarkably, the investigation of the behavior of micellar solutions and microemulsions with the aim of extracting a solute in a centrifugal field away from proximity to a CP has been ignored in the last 20 years.

This is because “soft” centrifugation (up to 3,000 g) and modern-day preparative ultracentrifugation (up to 600,000 g) are generally believed to handle only low yields, and AUC is confined to the field of analytical chemistry. Emulsions such as micelles in milk show slow sedimentation speeds and ultralow concentration profiles compared to nanoparticles and clays. Since the seminal papers by Hildebrand et al. [4], Winnick et al. [5], and Onuki et al. [6], there is an abundant literature of centrifugation in complex fluids near the CP, but our aim is to understand centrifugation of fluids far from the CP.

Only 3 quantitative papers have been published in microemulsions: Hwan et al. [7] concluded that phase boundaries should move upon centrifugation. Later, Dvolaitzky et al. [8] concluded from AUC studies that microemulsions can be described as monodisperse spheres, a conclusion that was shown to be wrong via small-angle scattering and nuclear magnetic resonance studies [9]. It is quite interesting to notice that the concept of “surfactant-free microemulsions” (SFMEs) was derived from careful AUC observations by Smith et al. [10] as early as 1977, but the correct physical principles of these observations that could not be explained by the standard flexible microemulsion model were only given in 2016, when the term “ultraflexible microemulsions (UFME)” was coined [11]. Until now, the only quantitative sedimentation study available in the literature is by Bulut et al. [12], who have shown that the Winsor II equilibrium can even split into 4 phases because of Earth’s gravity field, i.e., without the need for centrifugal fields. Following Jean Perrin in his Nobel Prize work on emulsions [13], this important method can even be used to measure molecular forces in such solutions.

In this work, we focus on the behavior of the system water/ethanol/ethyl acetate under centrifugation. This ternary system is highly used because it allows efficient and selective extraction of lipids from biological materials without any membrane–protein or osmolytes [14] and without using toxic materials such as chloroform or methanol. The mixing thermodynamics of this system is well approximated by regular solution theory, and therefore, distinct aggregation as it is observed in the case of SFMEs is not present. We study this system with 6 different compositions. Their locations in the phase diagram are described as:

• Pre-Ouzo (PO10 and PO20): The region in which a pre-Ouzo type of aggregation occurs in SFMEs, the region close to the 2-phase region on the water-rich side, where the PO20 composition has more ethanol (20%) than the PO10 composition. The PO20 composition is the formulation used for flavored alcoholic drinks, while many of the effects in centrifugation are more pronounced at lower concentration of ethanol since the supersaturation of the immiscible solvents is higher.
• The dilute pre-Ouzo (dPO) point is a point similar to PO, but heavily used in practice, because of preconcentration of any hydrophobic solute. This is the region where dispersive liquid–liquid microextraction (DLLME) [15] is performed.
• In the region close to the CP, prominent critical fluctuations give rise to centrifuge-induced criticality (CIC). This type of fluctuation is already present in binary systems [2].
• The living network (LN) region is the solvent-rich region close to the phase boundary. This region has been investigated by scattering and molecular dynamics. This region is the domain where the elusive “reverse” Ouzo effect was reported initially by Vitale et al. [16] but not yet quantified to our best knowledge. The microstructure is best understood as a 3-dimensional (3D) dynamical network of domains connected by hydrogen bonds [17]. The connection points in this network are the sites where excess water nucleation can occur.
• The concentrated living network region CLN is the intermediate domain between the CP and the LN region. It can be unambiguously quantified by scattering since the dominant scattering is a broad Lorentzian peak corresponding to a network mesh size and, at the same time, a smaller contribution as an Ornstein–Zernike function due to the proximity of the CP: The fluctuation can be understood as water molecules nucleating at cross-link points of the connected hydrogen bond network [18].
• Maceration point (MP): The point high up in the phase diagram that is used for maceration in case of extraction of organic molecules from plants. This hydrotrope-rich region in which no local structuration occurs is also used in the broadly used “Bligh and Dyer” methods and their greener version [14].

We report in this work the existence of 2 different types of phase separation induced by centrifugation: Close to the CP, CIC is induced similar to the already reported binary case [2]. However, in the ternary case, far from the CP, not only clouding occurs but also a new phenomenon of centrifuge-induced emulsification (CIE) leading to efficient creaming is observed. The experimental observations are rationalized with the help our recently developed centrifugation map (CMap) theory [19].

## Experimental Results

**Fig. 1.**
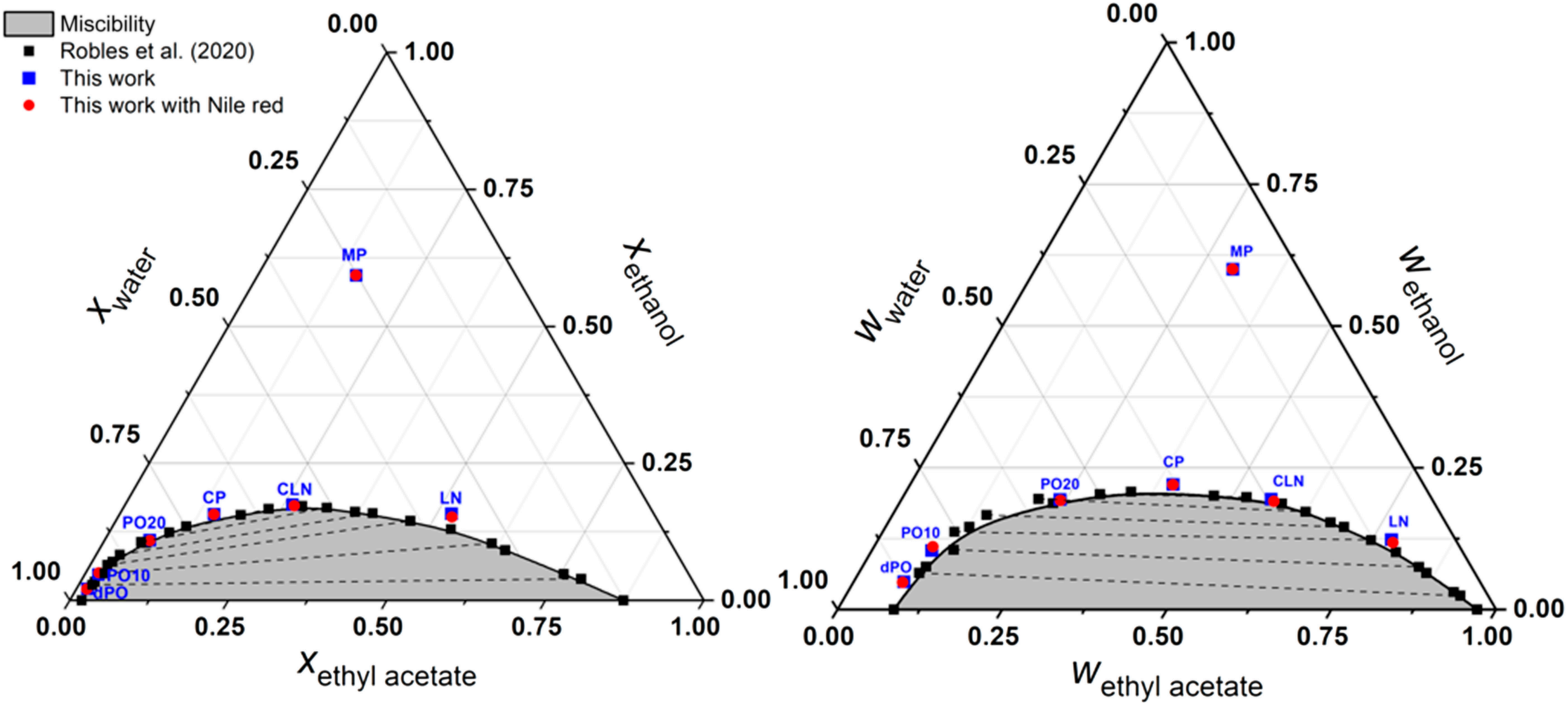
Ternary phase diagram of ethyl acetate/ethanol/water by mole (left) and weight (right) fraction, showing the miscibility gap and the tie lines determined by Robles et al. [21] (black cubes). Experimental points obtained in this work without Nile red (blue cubes) and with Nile red (red spheres) at 25 °C and atmospheric pressure are shown. (For interpretation of the references to color in this figure legend, the reader is referred to the web version of this article.)

In Fig. 1, we show the ternary phase diagram. Literature data from Robles et al. [21] are shown along with our own measurements with and without Nile red dye. The good alignment of the red points (with dye) with the other data points shows that the presence of Nile red has no influence on the phase behavior of the mixture. The plot in weight fraction scale indicates that there are no heterophase fluctuations [32] present because the CP is highly symmetric, while the representation in mole fraction indicates from the slopes of tie lines that the free energy of transfer at constant mole fraction between water rich and water poor is below 1 *k*_B_*T*.

**Fig. 2.**
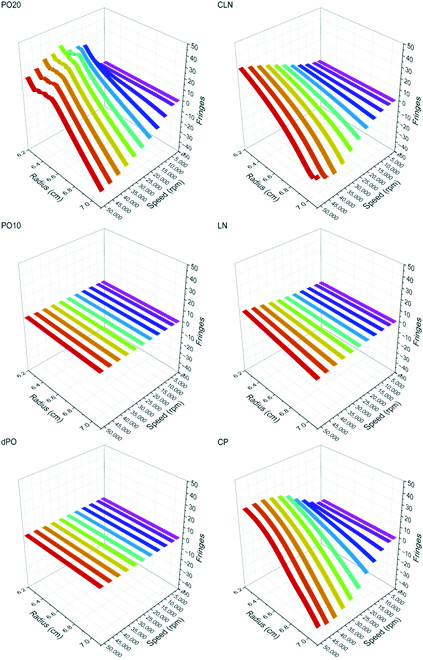
Raw data of sedimentation equilibria as number of fringes *∆J*(*r*) versus radius and different experimental speeds for the 6 studied ternary compositions. No Nile red as dye is present, and the temperature is 25 °C. (For interpretation of the references to color in this figure legend, the reader is referred to the web version of this article.)

**Fig. 3.**
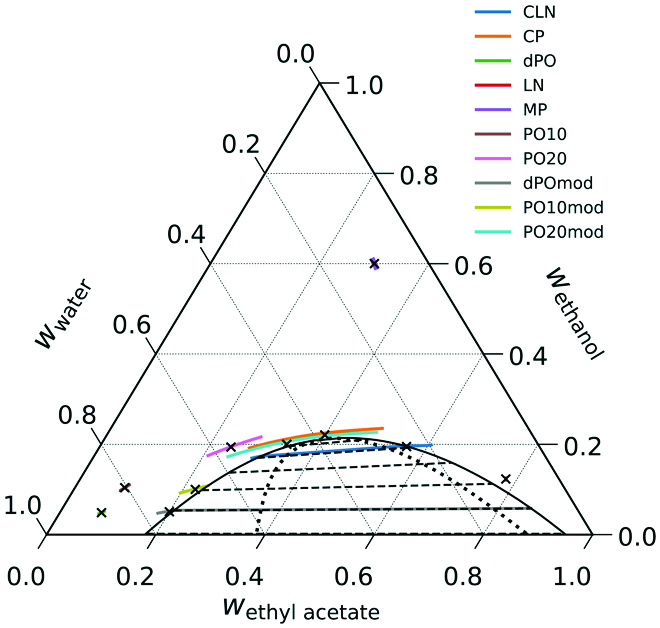
Theoretically predicted phase diagram and theoretical predictions for the 50,000 rpm composition profiles for the points studied by theory. The lines present the expected composition profiles in a real centrifugation cell between 6.2 and 7.2 cm. The densest, water-rich compositions appear on the left and bottom of the curves. The point MP, which is used for maceration in practice, is insensitive to centrifugal fields because hydrotrope and solvent have almost the same density.

Fig. 2 shows the raw centrifugation results for the different points as the number of fringes as a function of radius and centrifugation speed (for further raw data also for samples containing Nile red, see Figs. S1 and S2). At the PO20 composition, CIE occurs at the top of the tube: Turbidity appears, and there is flotation of water-poor droplets that coalesce and form a thin layer of water-poor phase at the top. In this situation, centrifugation can be understood as a similar effect as dilution by water, which induces the well-known Ouzo effect [16]. In contrast, the dPO and PO10 compositions show no such emulsification, because the nonpolar components are too dilute for this to happen at the used speed. The explanation follows in Fig. 3. For the CLN composition, we observe “reverse Ouzo” emulsification upon centrifugation, where a water-rich layer at the bottom of the tube appears: Water-rich droplets are formed and quickly sediment to the bottom of the tube.

For the LN point, the system is again too dilute, and emulsification will only occur at higher speeds*.* At the CP, CIC is observed. This is not strictly a phase transition, where 2 phases are separated by a thin interfacial layer, but a continuous change of composition that becomes practically indistinguishable from a true phase separation at high centrifugation speed.

**Fig. 4.**
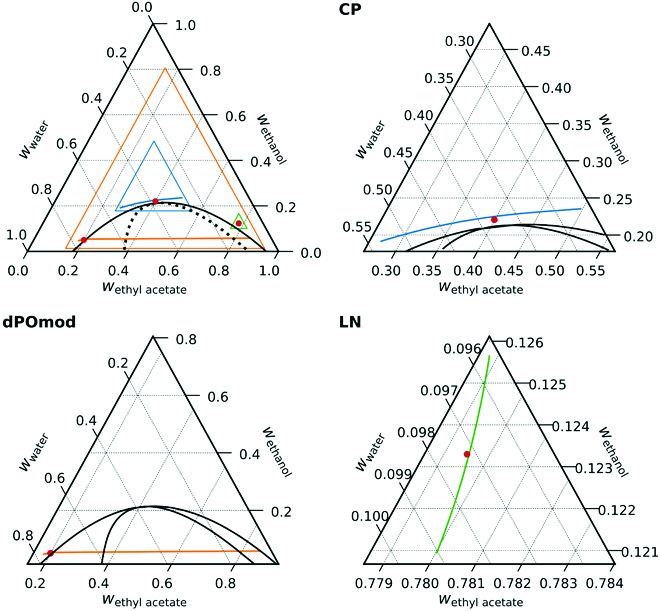
Full-phase diagram (top left) and enlarged composition profile plots for the points critical point (CP) in blue, dPO in orange, and LN in green. Near the CP, the system is extremely susceptible to external fields, because large-amplitude density fluctuations are present. In the dilute pre-Ouzo region, where the point dPO is located, spontaneous emulsification results in a large shift in composition across the miscibility gap. (For interpretation of the references to color in this figure legend, the reader is referred to the web version of this article.)

In Fig. 3, the theoretically calculated phase diagram is shown along with the CMap predictions for the composition profile in a centrifugation experiment at 50,000 rpm for the 6 points where measurements are taken (see Fig. 3). First, we note that the phase diagram is qualitatively well reproduced by our model, but the UNIQUAC model fails to quantitatively describe the phase stability. The left binodal is shifted inward relative to the experimental phase diagram. Thus, the dPO, PO10, and PO20 points lie rather far from the binodal. Theoretical calculations at these compositions poorly relate to the experiments, where the composition is close to the binodal, and CIE can show up. The compositions dPO, PO10, and PO20 are far from the phase boundary, and thus, the CMap theory for these points does not predict a crossing of the 2-phase region. For a better comparison of the experiments that were done at these 3 points, we introduce modified compositions dPOmod, PO10mod, and PO20mod: These compositions are relatively close to the theoretical phase boundary in locations that compare well to the respective positions of dPO, PO10, and PO20 relative to the experimental phase boundary. Calculations using these compositions thus relate to the situation in the experiments, and the theoretical prediction at 50,000 rpm is that CIE occurs, which agrees well with experiment. Fig. 4 shows the theoretical centrifugation profiles for 3 of these points: CP, dPOmod, and LN at larger magnification. The bent curve at the CP composition that is already visible in Fig. 3 is evident. Even at the LN point, where no composition changes in the tube are visible in Fig. 3, the magnified plot reveals a distinct, slightly bent profile. The dPOmod composition is close to the phase boundary and crosses the 2-phase region.

Fig. 4 shows 3 composition profiles in more detail. There are profound differences between the behavior near the CP shown, where the line connecting all local compositions is shown in blue, and the one that occurs at low concentration near the pre-Ouzo region, which is shown in orange. The line near the CP does not cross any binodal or the spinodal lines: No phase separation occurs; therefore, there is no nucleation and growth of a distinct phase. In the case shown in orange, the turbidity that appears at the top of the phase is the subject of coalescence and finally forms a light clear phase of ethyl acetate-rich phase at the top of the tube. The expected behavior observed near the LN point is shown in green: The scale is enlarged since the variation in composition is expected to be small. In this case, the introduction of a phase nucleation at the bottom of the tube is also possible, if the initial LN composition is located extremely close to the binodal line.

**Fig. 5.**
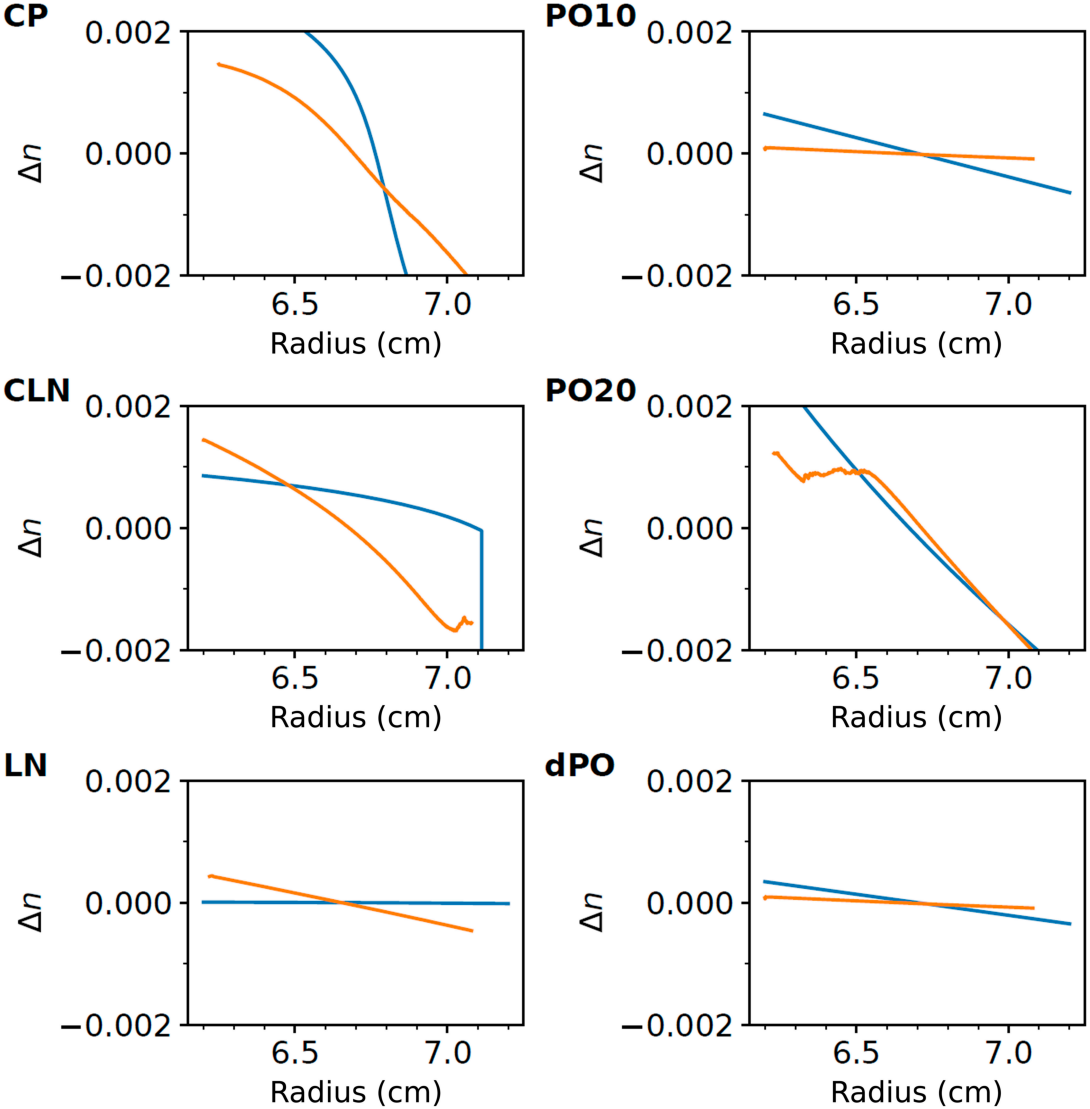
Comparison between experimental (orange) and predicted (blue) refractive index changes ∆*n* for the points CP, PO10, CLN, PO20, LN, and dPO. Note that the theoretical predictions for dPO, PO10, and PO20 are based on the points dPOmod, PO10mod, and PO20mod. (For interpretation of the references to color in this figure legend, the reader is referred to the web version of this article.)

Fig. 5 shows a quantitative comparison of experimental and theoretical results. Profiles of the index of refraction for centrifugation at 50,000 rpm are shown. The experimental data are derived from the number of fringe raw data, and the theoretical curves are derived from the predicted composition profiles and the fitted refractive index. The general trend is that the measured index of refraction goes down with increasing radius unless turbid zones are encountered, which result in noisy lines. This is seen for the points CLN and PO20. The different behavior of the theoretical prediction for point PO20 is easily explained: PO20 on the water-rich side of the phase diagram is farther away from the binodal line in the theoretical phase diagram than it is in experiments, and thus, the 2-phase region is not entered. For high contents of ethyl acetate, the experimental phase diagram is well reproduced by theory, and consequently, phase separation is predicted for the composition CLN just at the outer end of the tube, similar to the actual experimental observation.

Overall, the index of refraction along the tube agrees well for all 6 data points. No adjustable parameters were needed to obtain this agreement, which demonstrates the predictive power of our CMap theory.

**Fig. 6.**
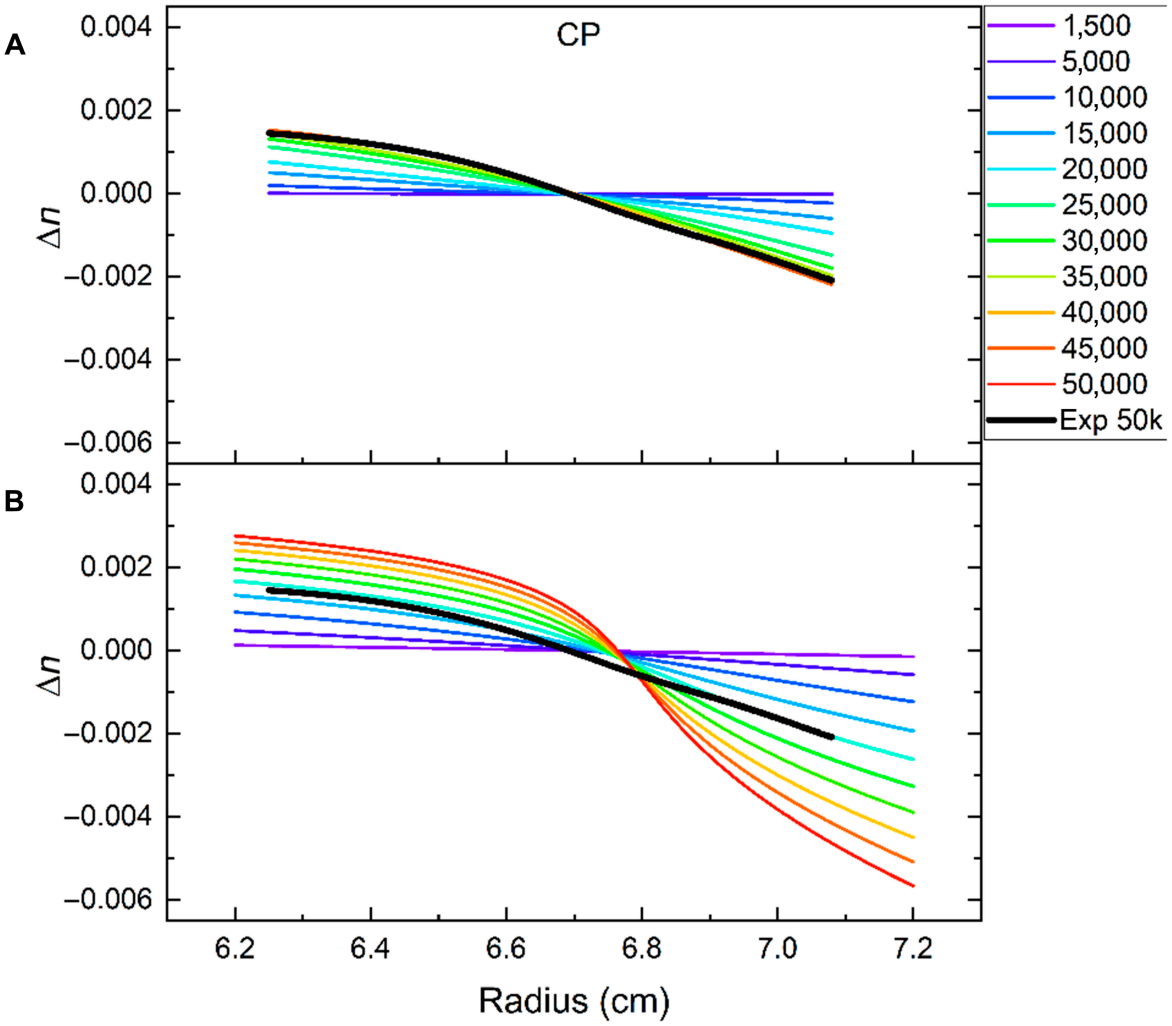
Comparison of the recalculated refractive index profile *∆n* versus radius for the CP experimental point from experimental sedimentation equilibrium data (A) and predicted refractive index changes (B) for different rotation speeds. In both plots, the thick black line is the experimental sedimentation equilibrium profile at 50,000 rpm (exp 50k).

In Fig. 6, we show the refractive index profile for the CP experiment and the CMap predictions for different speeds up to 50,000 rpm. The quantitative comparison reveals that the CMap prediction overestimates the refractive index change in the central region, while the slopes to the left and right region agree much better. The strong drop-in refractive index between 6.6 and 7.0 cm is an artifact, which arises close to the CP. The refractive index gradients at different speeds for the other samples with and without Nile red are shown in Figs. S3 to S5 as a 3D plot or in Figs. S6 and S7 as 2D representations.

**Fig. 7.**
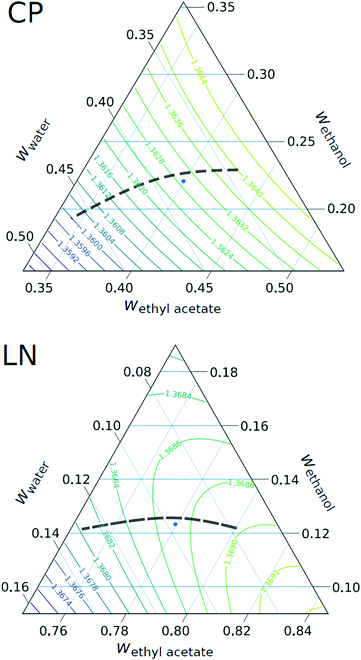
Contour plot of the refractive index in the region of the CP and the LN composition. The black dashed lines show a prediction of a possible composition profile based on experimental refractive index measurements and theoretical composition gradients.

So far, we have shown CMap predictions for the composition profiles in the ternary phase diagram. The reverse calculation of the refractive index allows mapping the refractive index profiles in a centrifugation cell, as shown in Fig. 6, onto the lines showing the composition profiles in the ternary phase diagram. This is shown in Fig. 7.

**Fig. 8.**
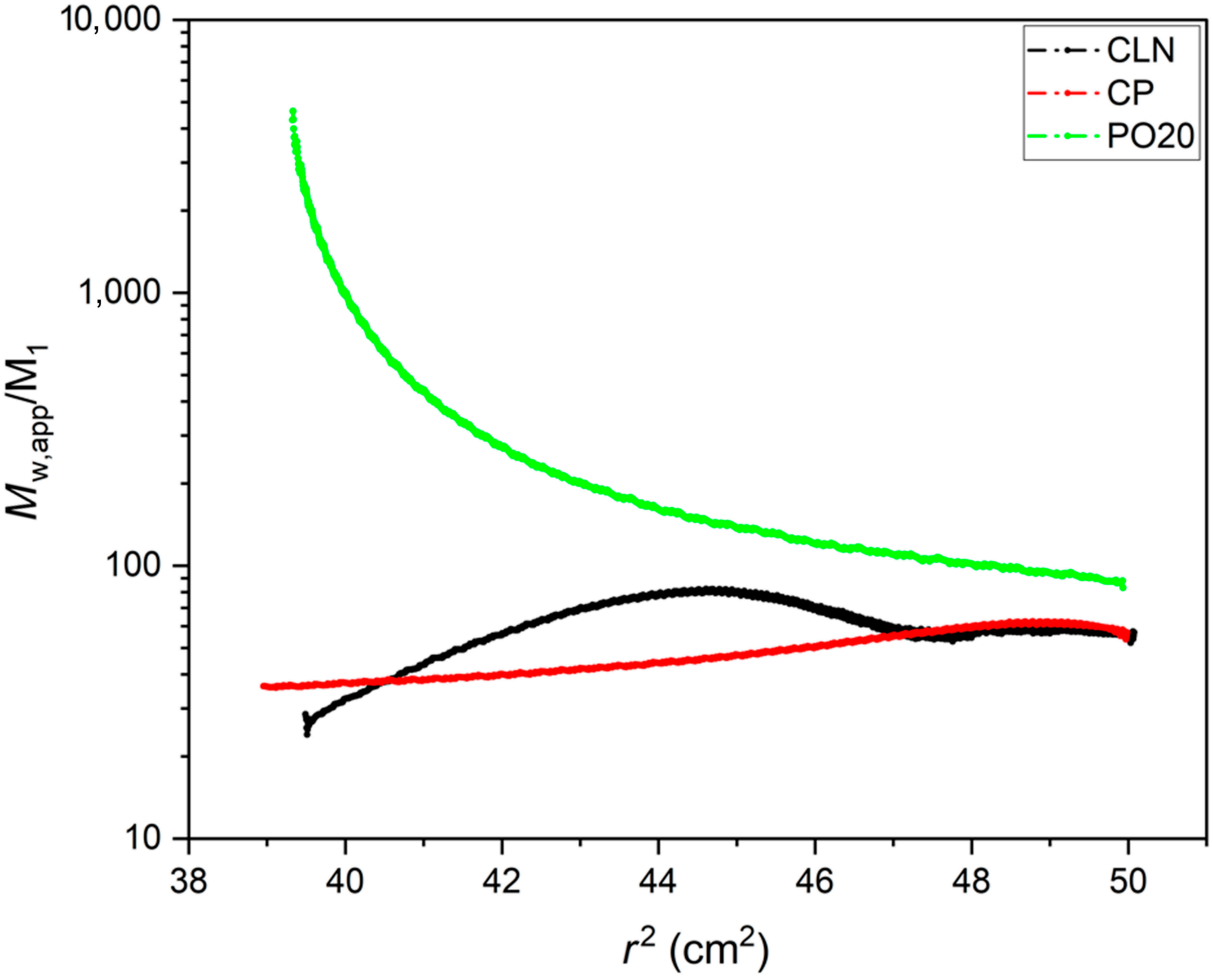
Reduced apparent weight–averaged molar mass profiles (*M*_w,app_/*M*_1_) as a function of *r*^2^ determined from flotation equilibrium experiments at 25 °C using Eq. 1. Red, CP at 50,000 rpm (190,000 g at 6.7 cm). The concentration gradients observed corresponds to an average apparent mass of 60 molecules. Black, point CLN at 40,000 rpm (120,000 g at 6.7 cm). A reduced apparent floating object mass of 47 is observed. Green, point PO20 in the pre-Ouzo region at 15,000 rpm (17,000 g at 6.7 cm): Aggregates are centrifuged as whole dynamic aggregates with an average content of 330 molecules. (For interpretation of the references to color in this figure legend, the reader is referred to the web version of this article.)

Finally, Fig. 8 shows the reduced apparent weight-averaged molar mass profiles (*M*_w,app_*/M*_1_) observed using the standard expression used for noninteracting spherical droplets, where *M*_w,app_ is given by:

$$M_{w,\mathrm{app}}\left(r\right)=\frac{1}{k}∙\frac{d\left(\text{ln}c\left(r\right)\right)}{d\left(r^{2}\right)}≅\frac{1}{k}∙\frac{d\left(\text{ln}\left[J_{a}+∆J\left(r\right)\right]\right)}{d\left(r^{2}\right)}$$ (1)

with *k* = (1 −*ρ*_0_/*ρ*_1_)*ω*^2^*/*2*RT*; *ρ*_0_ and *ρ*_1_ are the solvent and solute density, respectively; *ω* is the angular velocity (2*π* rpm/60); *R* is the gas constant; *T* the thermodynamic temperature; *c* is the concentration; *J_a_* is the absolute number of fringes at the meniscus for the case of sedimentation equilibrium; accordingly, at the bottom for the case of flotation equilibrium, ∆*J*(*r*) is the relative radial fringe shift (measured number of fringes with the offset to the meniscus); and *r* is the radial distance from the center of rotation. For the calculation of the reduced apparent molar mass, the values used are listed in Table S1.

As can be seen, near the phase boundary, the efficiency of centrifugation is enhanced by a small factor (<10) near the CP, while it is enhanced in the connected network structure by a larger factor, close to 50. It is extreme in the pre-Ouzo region, >100. As a consequence, in this region, centrifugation speeds of 15,000 rpm acting on pre-Ouzo aggregates have the same separation factor as 50,000 rpm (hundred times the centrifugal acceleration) that are only possible for small volumes.

## Methods

### Chemicals

Ethanol (for spectroscopy; Uvasol, purity ≥ 99.9%) and ethyl acetate (for spectroscopy; Uvasol, purity ≥ 99.9%) were purchased from Merck KGaA (Darmstadt, Germany). Nile red (technical grade) was purchased from Sigma-Aldrich Chemie GmbH (Steinheim, Germany). Water was Milli-Q quality with an organic total mass of 2 ppb (parts per billion) and resistivity of 18.2 MΩ/cm. All chemicals were used without further purification. The literature values and measured values of the refractive indices, densities, and viscosities of the 3 pure components are presented in Table S2. Figs. S8 and S9 show the literature densities and refractive index map of the ternary system at 25 °C, and Fig. S10 shows the modeled refractive index map. The description of the used software and fitting parameters needed for the calculations and visualization of the refractive index map can be found in the Supplementary Information and Table S3. The relative errors of the calculated refractive index at 25 °C compared to the combined experimental values of Andrade et al. [20] and Robles et al. [21] are shown in Fig. S11. These errors are in the fourth digit of the refractive index.

### Analytical ultracentrifugation

For the measurements, the ternary mixtures were prepared by weighing masses of about 1 g to ±0.1 mg on an analytical balance (ED224S, Sartorius AG Germany). The samples were prepared in screw thread vials (10 ml, 55 × 20 mm, clear glass) with a screw cap DIN 18. Ethanol was added dropwise with a 200-μl micropipette (Finnpipette F1) to the ethyl acetate/water binary mixture until the turbid solution became clear. For the samples with Nile red, a 0.0261 mM ethanol dye stock solution was prepared. Fig. 1 shows the mass fraction points of the sample mixtures that were selected from the ternary phase diagram. The exact compositions of the mixtures are listed in Table S4.

The measurements were performed on a modified Optima XL 80k (Beckman Coulter, Palo Alto, CA, USA) using an ultraviolet/visible (UV/Vis) multiwavelength (MWL) detector [22] and an advanced Rayleigh interference optics developed by Nanolytics [23]. A 12-mm double-sector titanium centerpiece was used with 340 μl of reference and sample solution. The general setup of the MWL detector is described in [22,24,25]. The raw data from the MWL detector are shown in Figs. S12 to S14, acquired at 50,000 rpm.

In the AUC, the interference optics is based on the principle of a Rayleigh interferometer as result of an interference pattern of parallel light and dark fringes behind the measurement cell. Such raw Rayleigh interference data are shown in Fig. S15 collected at 50,000 rpm. At each radial position (*r*), the pattern of interference fringe reflects differences in the refractive index, *∆n*(*r*), between the solution and the solvent. The experimentally obtained radial interference fringe shifts is the result of a Fourier transform analysis [26] of the experimental interference fringe pattern. This vertical shift of the interference fringes is counted in numbers of fringes, *∆J*(*r*):

$$∆J\left(r\right)=\frac{∆n\left(r\right)*a}{\lambda }$$ (2)

where *a* is the thickness of the centerpiece (1.2 cm), *λ* is the wavelength of the light (675 nm), and *Δn*(*r*) is the refractive index difference, *∆n*(*r*) = *n*_solution_(*r*) − *n*_solvent_(*r*). More theory of the calculations using the experimental fringes is described in our work on binary mixtures [2].

The speed variation experiments were performed at a constant temperature of 25 °C (±0.1 °C) with speeds between 1,500 and 50,000 rpm. Fig. 2 and Figs. S1 and S2 show the fringe shifts *∆J*(*r*) in dependence of different experimental speeds. The fringe shift is 0 at the point of the original composition. All raw data were presented using OriginLab 2020 [27] and a self-written LabVIEW [28] program.

### Theory

The theoretical prediction of the system follows the procedure of our recent work [19], where also further details of the derivation are given. The free energy of the system is a functional of the particle densities {*ρ_i_*(**r**)} in the framework of density functional theory [29–31]. Within the local density approximation, for which nonlocal correlations are neglected, the free energy of an inhomogeneous system can be written as:

$$F[\{\rho_{i}(r)\}]=∭f(\{\rho_{i}(r)\}]dr+∭\sum_{i}m_{i}\rho_{i}(r)\psi_{G}(r)dr$$ (3)

where *f*({*ρ_i_*(**r**)}) is the free energy per unit of volume, which can be expressed as *f*({*ρ_i_*(**r**)}) = ∑*_i_μ_i_ρ_i_*(**r**) − *P* with the chemical potential *μ_i_* and the pressure *P* for a fixed temperature *T*. Following minimization of the functional with Lagrange multipliers and multiple steps of simplification, we showed that, in equilibrium, composition profiles follow:

$$k_{B}T{\left(\frac{\partial }{\partial r}\right)}_{P}\text{ln}\left(x_{i}\gamma_{i}\right)-\left(m_{i}-\frac{V_{i}\sum_{j}x_{j}m_{j}}{\sum_{k}x_{k}V_{k}}\right)g=0$$ (4)

Here, *k*_B_ is the Boltzmann constant and *g* the gravitational acceleration (in the case of centrifugation *g* = *ω*^2^*r*). Furthermore, *x_i_*, *γ_i_*, *m_i_*, and *V_i_* denote the mole fraction, activity coefficient, (molecular) mass, and partial volume of component *i*, respectively. Having an appropriate model for the activity coefficients, the composition profile can be calculated numerically from this equation. Here, we use an UNIQUAC activity coefficient model similar to our previous work [19]. This model gives a good representation of the mixing thermodynamics. Practically, a starting composition at *r*_0_ has to be set and then propagated throughout the vial in adequate steps of d*r*. At equilibrium, the composition at *r* + d*r* follows:

$$\begin{array}{c} k_{B}T\text{ln}x_{i}\left(r+dr\right)+k_{B}T\text{ln}\gamma_{i}\left(\left\{x\right\}\left(r+dr\right)\right)-\\ k_{B}T\text{ln}x_{i}\left(r\right)+k_{B}T\text{ln}\gamma_{i}\left(\left\{x\right\}\left(r\right)\right)-m_{i}^{\prime }gdr=0 \end{array}$$ (5)

where $m_{i}^{\prime }$ is the buoyancy-corrected mass. It should be noted that Eq. 4 has to be solved only for 2 independent components. The sedimentation length *l*_sed_ is first estimated as the respective ideal value:

$$l_{\mathrm{sed}}=\frac{k_{B}T}{\omega^{2}r_{\max}m_{i}^{\prime }}$$ (6)

for all components *i*. After solving the equations step by step for all *r*, the overall composition in the vial is evaluated. The step size d*r* is then set to the minimum sedimentation length (or the vial height, whatever happens to be smaller) divided by 1,000. By repeatedly optimizing the initial composition at *r*_0_, the overall composition ⟨*x*⟩ converges to a desired value.

## Conclusion and Outlook

In this work, we have shown and described centrifugation of 3-component liquid mixtures by experiments (based on refractive index profiles) and theory. We characterize 2 types of phase separation in these systems: CIC and CIE. While CIE is a true phase transition in which there is a sudden jump in composition with a molecular-scale interfacial width, in CIC, the composition changes are gradual but practically appear as very steep concentration gradients when the centrifugal field is high. Away from the region where the transition occurs, the composition gradients are small, as one would expect for a mixture of simple liquids.

Addition of a dye like Nile red has 2 advantages. First, it has no influence on the phase diagram, and the data with or without Nile red are identical. Second, using UV/Vis absorption optics, it allows for the possibility to determine the Nile red concentration as a model solute to demonstrate the separation capability of our ternary solvent system in a centrifugal field.

This work lays out a reference for the various phenomena that centrifugation can induce in liquid mixtures. The system studied in this work does not comprise substantial aggregation. The next step will be an extension to aggregating systems and even colloidal systems like micelles or microemulsions, for which a large variety of effects can be expected.

Finally, the plot of an apparent sedimenting particle molar mass in Fig. 8 reveals the high potential of centrifugation near-phase boundaries between a 2-phase region and a monophasic region that can be described as an ultraflexible microemulsion. When the apparent mass is derived from the strong gradients obtained in the “pre-Ouzo” PO region, this corresponds to an amplification of a factor of 60 versus isolated molecules. In terms of centrifugal acceleration, this allows a reduction of a factor of 20, which allows continuous preparative “soft” centrifugation. The well-studied centrifugation near the CP or the sedimentation and coalescence observed starting from the solvent-rich corner (CLN) are much less effective, even above 40,000 rpm.

The high sensitivity in a region where ternary aggregates that can be described as emerging micelles form is the source of the outstanding efficiency of preconcentration and analytic extraction with high efficiency whatever the hydrophobicity (log *P*) of the trace solute to be analyzed is. The DLLME method was proposed in 2006 [15] and has expanded very quickly as a reliable method to extract analytes with soft sedimentation, used for a large panel of analytical problems [33]. It is likely that the similarity of water/ethanol/ethyl acetate to systems like octanol/ethanol/water is high enough for the identification of the processes that occur between the binodal and spinodal lines near the phase boundary, where also ultraflexible microemulsions form. Our study allows proposing a first understanding of the molecular driving forces responsible for the DLLME efficiency.

## Data Availability

The experimental data are available upon reasonable request from R.R., D.H., and H.C.

## Supplementary Materials

Supplementary Materials Table S1. Molar mass (*M*_1_), densities of sedimenting macromolecule (*ρ*_1_) and solvent mixture (*ρ*_0_), and absolute number of fringes at the bottom of cell (*J_a_*), which are necessary for the calculation of the apparent weight-averaged molar mass (*M*_w,app_/*M*_1_) for experimental points PO20, CP, and CLN at 25 °C. Table S2. Molar mass (*M*_1_), refractive indices (*n*), densities (*ρ*), viscosities (*η*), and relative dielectric constants (*ε*) for pure liquids at 25 °C. Table S3. Fitting parameters for the binary RK expressions (Eq. S3) and the ternary deviation (Eq. S4). Table S4. Experimental compositions (mass, *w* and mole, *x* fraction) of the ethyl acetate (EA)/ethanol (EtOH)/water (H_2_O) mixtures and the reference solutions at 25 °C and atmospheric pressure. Fig. S1. 3D plot of raw interference data of flotation equilibria measured as number of fringes *∆J*(*r*) versus radial distance of the measured cell from the rotor centrum (radius, *r*) and different speeds for the experimental points for the ternary system **with** the presence of Nile red (0.0261 mM ethanol dye stock solution) at 25 °C. Fig. S2. 3D plot of raw interference data of flotation equilibria measured as number of fringes *∆J*(*r*) versus radial distance of the measured cell from the rotor centrum (radius, *r*) and different speeds for the reference experimental point MP for the ethyl acetate/ethanol/water ternary system and CRM for the *n*-octanol/ethanol/water ternary system with and without the presence of Nile red at 25 °C. Fig. S3. From interference data of sedimentation equilibria, the recalculated refractive index profile *∆n* versus radial distance of the measured cell from the rotor centrum (radius, *r*) and different speeds for the CP, CLN, and LN experimental points for the ethyl acetate/ethanol/water ternary system without (left) and with (right) Nile red as dye at 25 °C. Fig. S4. Continuation of Fig. S3 for the experimental points PO20, PO10, and dPO. For caption, see Fig. S3. Fig. S5. Comparison of a nearly regular solution and a system with pre-Ouzo effect: From interference data of flotation equilibria, the recalculated refractive index profile *∆n* versus radial distance of the measured cell from the rotor centrum (radius, *r*) and different speeds for the MP experimental point for the ethyl acetate/ethanol/water ternary system and the CRM experimental point for the *n*-dodecane/ethanol/water ternary system with (right) and without (left) Nile red at 25 °C. Fig. S6. Refractive index profile *∆n* recalculated from the interference fringe versus radial distance of the measured cell from the rotor centrum (radius, *r*) as 2D plot. Fig. S7. Refractive index *n* recalculated from the experimental *∆n* (see Fig. S6) versus radial distance of the measured cell from the rotor centrum (radius, *r*). Fig. S8. Densities of the ethyl acetate/ethanol/water ternary system at 25 °C obtained by Pires et al. [10] (red spheres). Fig. S9. Refractive indices *n* of the ethyl acetate/ethanol/water ternary system at 25 °C obtained by Andrate et al. [7] (blue spheres) and Robles et al. [11] (red spheres). Fig. S10. Modeled refractive index *n* in the ethyl acetate/ethanol/water ternary system at 25 °C. Fig. S11. Relative error of calculated refractive index at 25 °C compared to the combined experimental values of Andrade et al. [7] and Robles et al. [11]. Fig. S12. MWL-UV/Vis raw data of sedimentation equilibria at 50,000 rpm and 25 °C for the experimental points for the ternary system ***with*** the presence of Nile red (0.0261 mM ethanol dye stock solution). Fig. S13. MWL-UV/Vis raw data of sedimentation equilibria at 50,000 rpm and 25 °C for the experimental points for the ternary system ***without*** the presence of Nile red. Fig. S14. MWL-UV/Vis raw data of sedimentation equilibria at 50,000 rpm and 25 °C for the reference experimental point MP for the ethyl acetate/ethanol/water ternary system and CRM for the *n*-octanol/ethanol/water ternary system with and without the presence of Nile red. Fig. S15. Raw images of the experimentally obtained interference fringe pattern for the ethyl acetate/ethanol/water ternary system at 50,000 rpm and 25 °C. Fig. S16. Polynomials of the second type for extrapolating the density of the binary solutions of (A) ethanol/water [12] and (B) ethyl acetate/ethanol [13] at 25 °C. Fig. S17. Images of the PO20-NR sample before and after centrifugation at 25 °C in the analytical ultracentrifuge at 50,000 rpm for 94 h. Supplementary Text.

## References

[1] The Nobel Prize in Chemistry 1926. NobelPrize.org. [accessed 21 Feb 2022] https://www.nobelprize.org/prizes/chemistry/1926/summary/.

[2] Zemb T, Rosenberg R, Marčelja S, Haffke D, Dufrêche J-F, Kunz W, Horinek D, Cölfen H. Phase separation of binary mixtures induced by soft centrifugal fields. Phys Chem Chem Phys. 2021;23(14):8261–8272. 33527947 10.1039/d0cp01527j

[3] Sicilia D, Rubio S, Pérez-Bendito D. Evaluation of the factors affecting extraction of organic compounds based on the acid-induced phase cloud point approach. Anal Chim Acta. 2002;460(1):13–22.

[4] Hildebrand JH, Alder BJ, Beams JW, Dixon HM. The effects of hydrostatic pressure and centrifugal fields upon critical liquid–liquid interfaces. J Phys Chem. 1954;58(8):577–579.

[5] Winnick J, Knobler CM, Scott RL. Critical phenomena in the ultracentrifuge: Some new experimental evidence. Physica A. 1989;156(1):77–91.

[6] Onuki A, Kitamura H. Solvation effects in near-critical binary mixtures. J Chem Phys. 2004;121(7):3143–3151. 15291624 10.1063/1.1769357

[7] Hwan R-N, Miller CA, Fort T. Determination of microemulsion phase continuity and drop size by ultracentrifugation. J Colloid Interface Sci. 1979;68(2):221–234.

[8] Dvolaitzky M, Guyot M, Lagües M, Le Pesant JP, Ober R, Sauterey C, Taupin C. A structural description of liquid particle dispersions: Ultracentrifugation and small angle neutron scattering studies of microemulsions. J Chem Phys. 1978;69(7):3279–3288.

[9] Gradzielski M, Duvail M, de Molina PM, Simon M, Talmon Y, Zemb T. Using microemulsions: Formulation based on knowledge of their mesostructure. Chem Rev. 2021;121(10):5671–5740. 33955731 10.1021/acs.chemrev.0c00812

[10] Smith GD, Donelan CE, Barden RE. Oil-continuous microemulsions composed of hexane, water, and 2-propanol. J Colloid Interface Sci. 1977;60(3):488–496.

[11] Zemb TN, Klossek M, Lopian T, Marcus J, Schöettl S, Horinek D, Prevost SF, Touraud D, Diat O, Marčelja S, et al. How to explain microemulsions formed by solvent mixtures without conventional surfactants. Proc Natl Acad Sci USA. 2016;113(16):4260–4265. 27044068 10.1073/pnas.1515708113 PMC4843454

[12] Bulut S, Åslund I, Topgaard D, Wennerström H, Olsson U. Lamellar phase separation in a centrifugal field. A method for measuring interbilayer forces. Soft Matter. 2010;6(18):4520.

[13] The Nobel Prize in Physics 1926. NobelPrize.org. [accessed 21 Feb 2022] https://www.nobelprize.org/prizes/physics/1926/summary/.

[14] Breil C, Abert Vian M, Zemb T, Kunz W, Chemat F. “Bligh and Dyer” and folch methods for solid–liquid–liquid extraction of lipids from microorganisms. comprehension of solvatation mechanisms and towards substitution with alternative solvents. Int J Mol Sci. 2017;18(4):708. 28346372 10.3390/ijms18040708 PMC5412294

[15] Rezaee M, Assadi Y, Milani Hosseini M-R, Aghaee E, Ahmadi F, Berijani S. Determination of organic compounds in water using dispersive liquid–liquid microextraction. J Chromatogr A. 2006;1116(1-2):1–9. 16574135 10.1016/j.chroma.2006.03.007

[16] Vitale SA, Katz JL. Liquid droplet dispersions formed by homogeneous liquid−liquid nucleation: “The ouzo effect”. Langmuir. 2003;19(10):4105–4110.

[17] Schöttl S, Lopian T, Prévost S, Touraud D, Grillo I, Diat O, Zemb T, Horinek D. Combined molecular dynamics (MD) and small angle scattering (SAS) analysis of organization on a nanometer-scale in ternary solvent solutions containing a hydrotrope. J Colloid Interface Sci. 2019;540:623–633. 30690387 10.1016/j.jcis.2019.01.037

[18] Roger K. Nanoemulsification in the vicinity of phase inversion: Disruption of bicontinuous structures in oil/surfactant/water systems. Curr Opin Colloid Interface Sci. 2016;25:120–128.

[19] Stemplinger S, Prévost S, Zemb T, Horinek D, Dufrêche J-F. Theory of ternary fluids under centrifugal fields. J Phys Chem B. 2021;125(43):12054–12062. 34694817 10.1021/acs.jpcb.1c05875

[20] Andrade RS, González C, Iglesias M. Changes of refractive indices for ethanol + water + (ethyl acetate or 1-pentanol) at 298.15 K. Int J Thermodyn. 2017;20(3):174–181.

[21] Robles PA, Lourenço NI, Igarashi EMS, Sousa MN, Arce PF.Thermodynamic behavior of the phase equilibrium of ethyl acetate + ethanol + water systems at atmospheric pressure: Experiment and modeling. J Chem Eng Data. 2020;65(4):1402–1410.

[22] Pearson J, Walter J, Peukert W, Cölfen H. Advanced multiwavelength detection in analytical ultracentrifugation. Anal Chem. 2018;90(2):1280–1291. 29214799 10.1021/acs.analchem.7b04056

[23] Nanolytics Instruments. Fine detectors and accessories for analytical ultracentrifugation. nanolytics-instruments.com. [accessed 21 Feb 2022] https://www.nanolytics-instruments.de/.

[24] Bhattacharyya SK, Maciejewska P, Börger L, Stadler M, Gülsün AM, Cicek HB, Cölfen H. Development of a fast fiber based UV-vis multiwavelength detector for an ultracentrifuge. In: Wandrey C, Cölfen H, editors. *Analytical ultracentrifugation VIII*. Berlin/Heidelberg (Germany): Springer-Verlag; 2006. Vol. 131, Progress in colloid and polymer science. pp. 9–22.

[25] Strauss HM, Karabudak E, Bhattacharyya S, Kretzschmar A, Wohlleben W, Cölfen H. Performance of a fast fiber based UV/Vis multiwavelength detector for the analytical ultracentrifuge. Colloid Polym Sci. 2008;286(2):121–128. 19816525 10.1007/s00396-007-1815-5 PMC2755732

[26] Yphantis DA, Lary JW, Stafford WF, Liu S, Olsen PH, Hayes DB, Moody TP, Ridgeway TM, Lyons DA, Laue TM, On line data acquisition for the Rayleigh interference optical system of the analytical ultracentrifuge. In: Schuster TM, Laue TM, editors. *Modern analytical ultracentrifugation: Acquisition and interpretation of data for biological and synthetic polymer systems*. 1st ed. Boston (MA): Birkhäuser; 1994. Emerging biochemical and biophysical techniques. pp 209–226.

[27] Origin 2019. [accessed 21 Feb 2022] https://www.originlab.com/index.aspx?go=Products/Origin/2019.

[28] LabVIEW. [accessed 21 Feb 2022] https://www.ni.com/en-us/shop/labview.html.

[29] Evans R. The nature of the liquid-vapour interface and other topics in the statistical mechanics of non-uniform. Classical Fluids Adv Phys. 1979;28(2):143–200.

[30] Henderson D. *Fundamentals of inhomogeneous fluids*. New York (NY): M. Dekker; 1992.

[31] Lutsko, J. F. Recent developments in classical density functional theory. In: Rice SA, editor. *Advances in chemical physics*. John Wiley & Sons; 2010. Vol. 144, Advances in chemical physics. pp. 1–92.

[32] Frenkel J. A general theory of heterophase fluctuations and pretransition phenomena. J Chem Phys. 1939;7(7):538–547.

[33] Rezaee M, Yamini Y, Faraji M. Evolution of dispersive liquid–liquid microextraction method. J Chromatogr A. 2010;1217(16):2342–2357. 20005521 10.1016/j.chroma.2009.11.088

