# Supplementary material for: Two Types of Liquid Phase Separation Induced by Soft Centrifugation in Aqueous Ethyl Acetate Using Ethanol as Cosolvent
Source: Research (Wash D C). 2023 Jan 16;6:0026. doi: 10.34133/research.0026 (PMC10076002; doi:10.34133/research.0026)
Supplement: Supplementary Materials — Table S1. Molar mass (M1), densities of sedimenting macromolecule (ρ1) and solvent mixture (ρ0), and absolute number of fringes at the bottom of cell (Ja), which are necessary for the calculation of the apparent weight-averaged molar mass (Mw,app/M1) for experimental points PO20, CP, and CLN at 25 °C. Table S2. Molar mass (M1), refractive indices (n), densities (ρ), viscosities (η), and relative dielectric constants (ε) for pure liquids at 25 °C. Table S3. Fitting parameters for the binary RK expressions (Eq. S3) and the ternary deviation (Eq. S4). Table S4. Experimental compositions (mass, w and mole, x fraction) of the ethyl acetate (EA)/ethanol (EtOH)/water (H2O) mixtures and the reference solutions at 25 °C and atmospheric pressure. Fig. S1. 3D plot of raw interference data of flotation equilibria measured as number of fringes ∆J(r) versus radial distance of the measured cell from the rotor centrum (radius, r) and different speeds for the experimental points for the ternary system with the presence of Nile red (0.0261 mM ethanol dye stock solution) at 25 °C. Fig. S2. 3D plot of raw interference data of flotation equilibria measured as number of fringes ∆J(r) versus radial distance of the measured cell from the rotor centrum (radius, r) and different speeds for the reference experimental point MP for the ethyl acetate/ethanol/water ternary system and CRM for the n-octanol/ethanol/water ternary system with and without the presence of Nile red at 25 °C. Fig. S3. From interference data of sedimentation equilibria, the recalculated refractive index profile ∆n versus radial distance of the measured cell from the rotor centrum (radius, r) and different speeds for the CP, CLN, and LN experimental points for the ethyl acetate/ethanol/water ternary system without (left) and with (right) Nile red as dye at 25 °C. Fig. S4. Continuation of Fig. S3 for the experimental points PO20, PO10, and dPO. For caption, see Fig. S3. Fig. S5. Comparison of a nearly regular solution and a [file research.0026.f1.pdf]

Supplementary Information

Two Types of Liquid Phase Separation Induced by Soft Centrifugation in Aqueous Ethyl Acetate Using Ethanol as Cosolvent

Helmut Cölfen[1], Rose Rosenberg[1], Dirk Haffke[1], Simon Stemplinger[2,3], Thomas Zemb[2], Dominik Horinek[3]

Affiliations

1 Physical Chemistry, Department of Chemistry, University of Konstanz, Universitätsstr. 10, D-78457 Konstanz

2 Institute for separation chemistry ICSM U Montpellier/CEA/CNRS/ENSCM, Marcoule, France

3 Institute of Physical and Theoretical Chemistry, University of Regensburg, D-93040 Regensburg

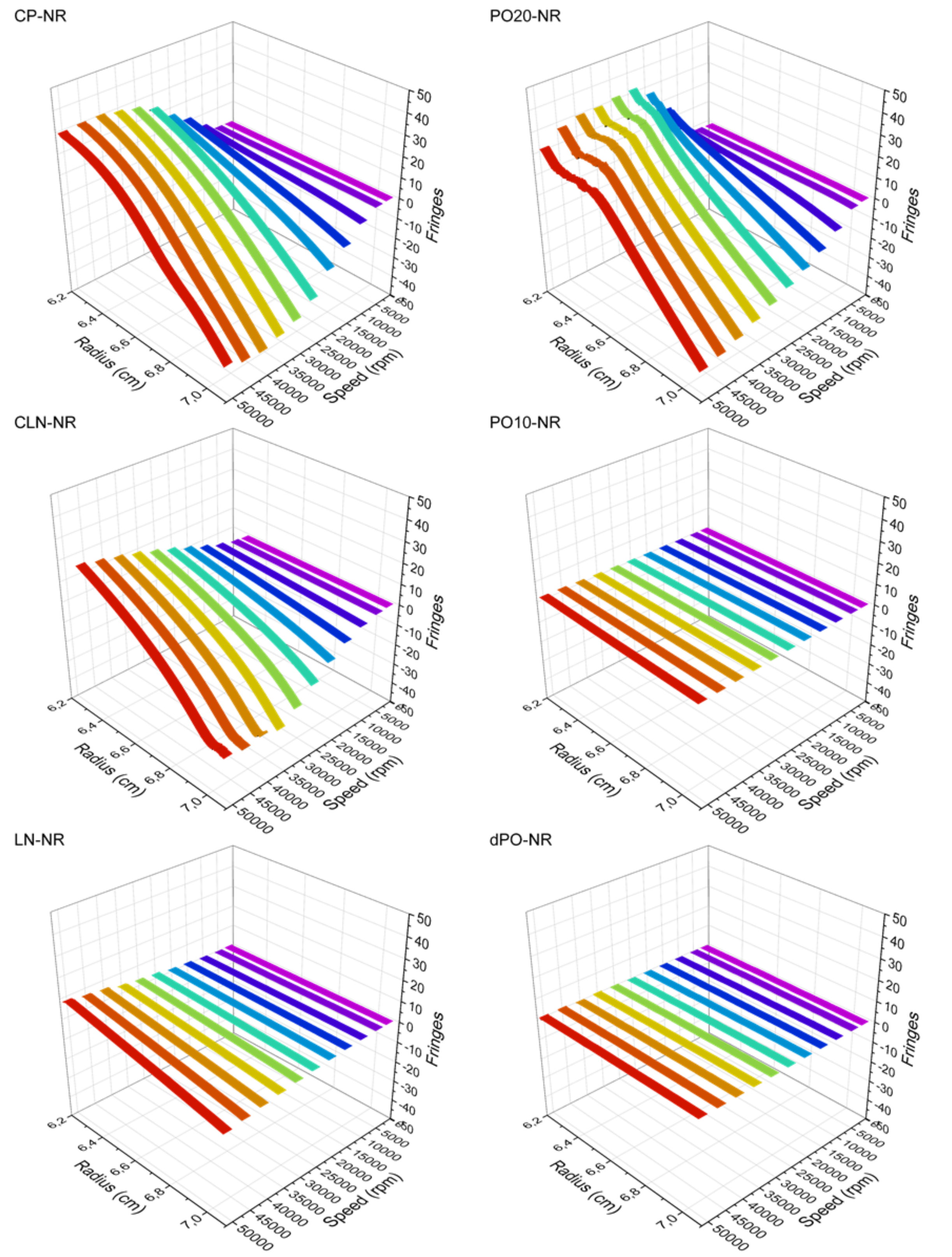

Figure S1. 3D plot of raw interference data of flotation equilibria measured as number of fringes Δ*J(r)* versus radial distance of the measured cell from the rotor centrum (radius, r) and different speeds for the experimental points for the ternary system **with** the presence of Nile red (0.0261 mM ethanol-dye stock solution) at 25 °C.

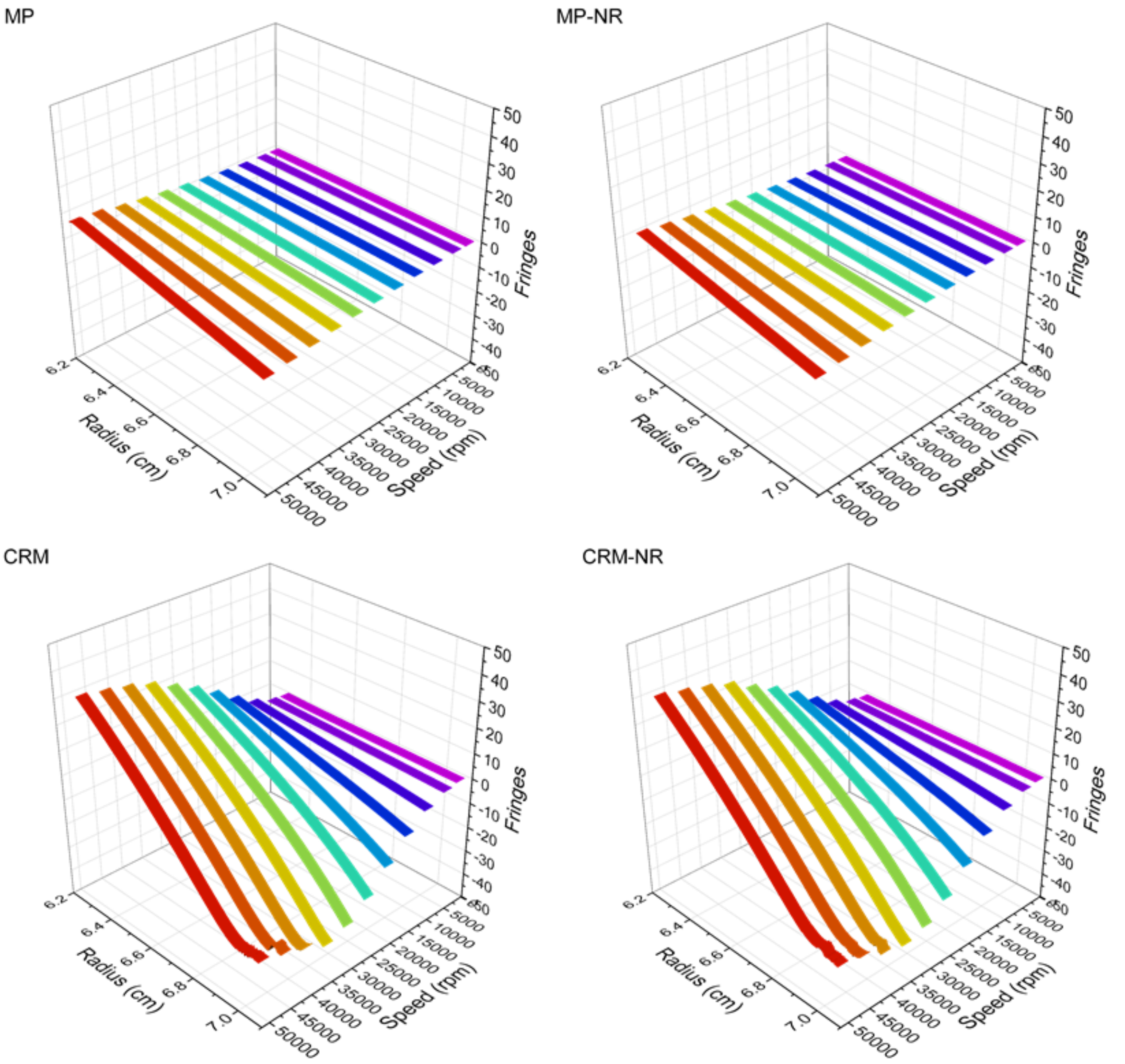

Figure S2. 3D plot of raw interference data of flotation equilibria measured as number of fringes Δ*J(r)* versus radial distance of the measured cell from the rotor centrum (radius, r) and different speeds for the reference experimental point MP for the ethyl acetate/ethanol/water ternary system and CRM for the *n*-octanol/ethanol/water ternary system with and without the presence of Nile red at 25 °C.

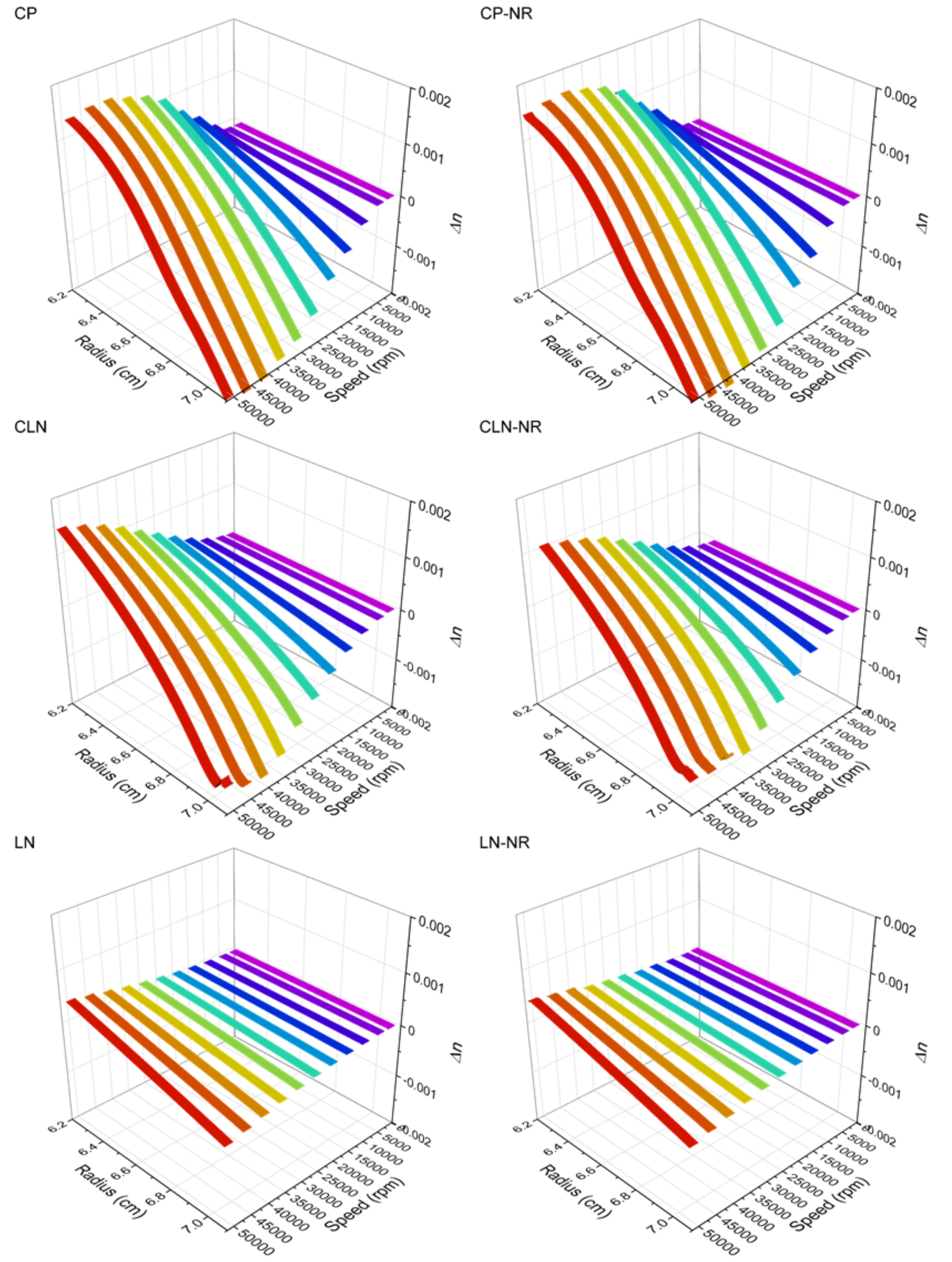

Figure S3. From interference data of sedimentation equilibria, the recalculated refractive index profile Δn versus radial distance of the measured cell from the rotor centrum (radius, r) and different speeds for the CP, CLN and LN experimental points for the ethyl acetate/ethanol/water ternary system without (left) and with (right) Nile red as dye at 25 °C.

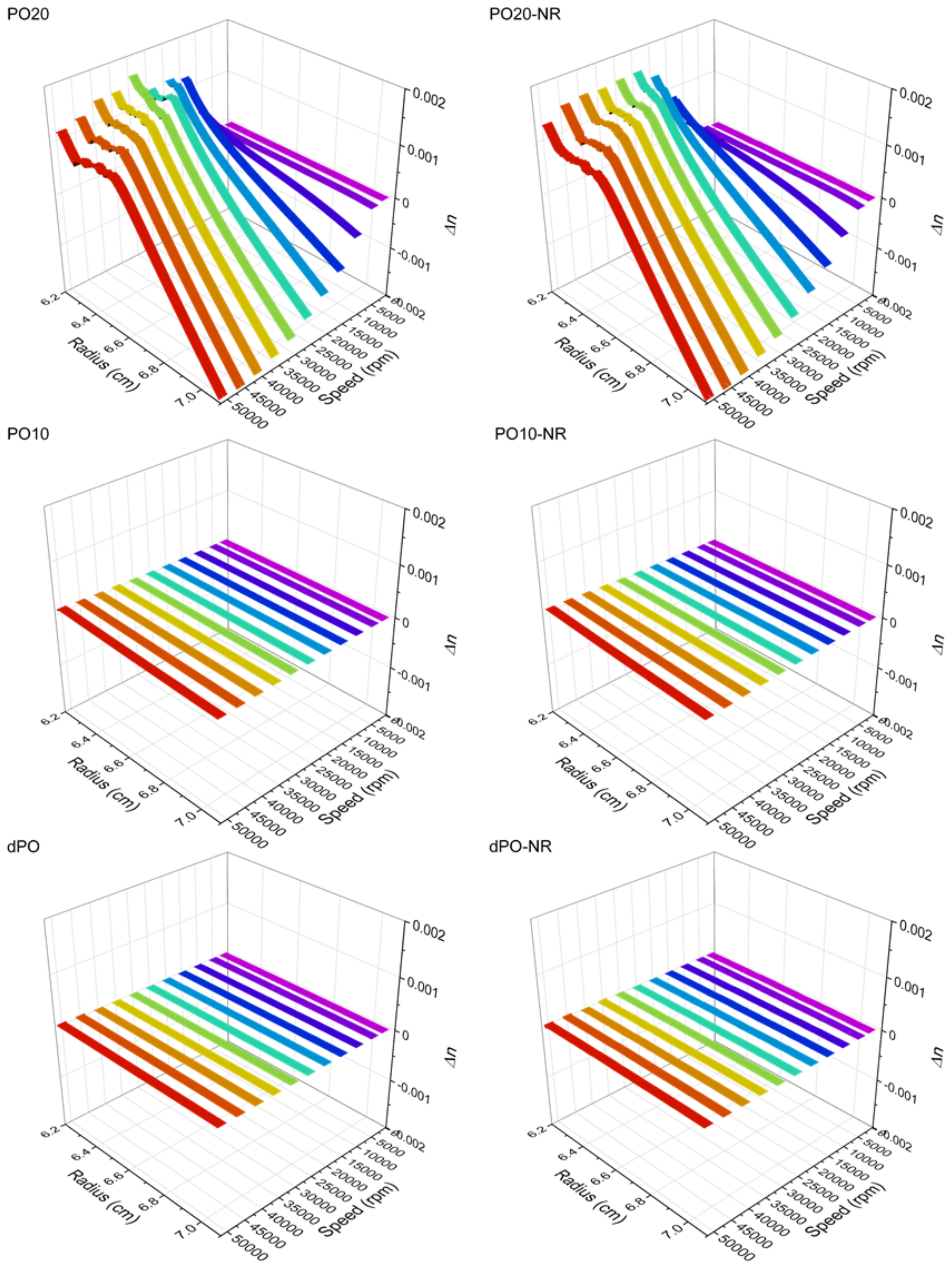

Figure S4. Continuation of Figure S3 for the experimental points PO20, PO10 and dPO. For caption, see Figure S3.

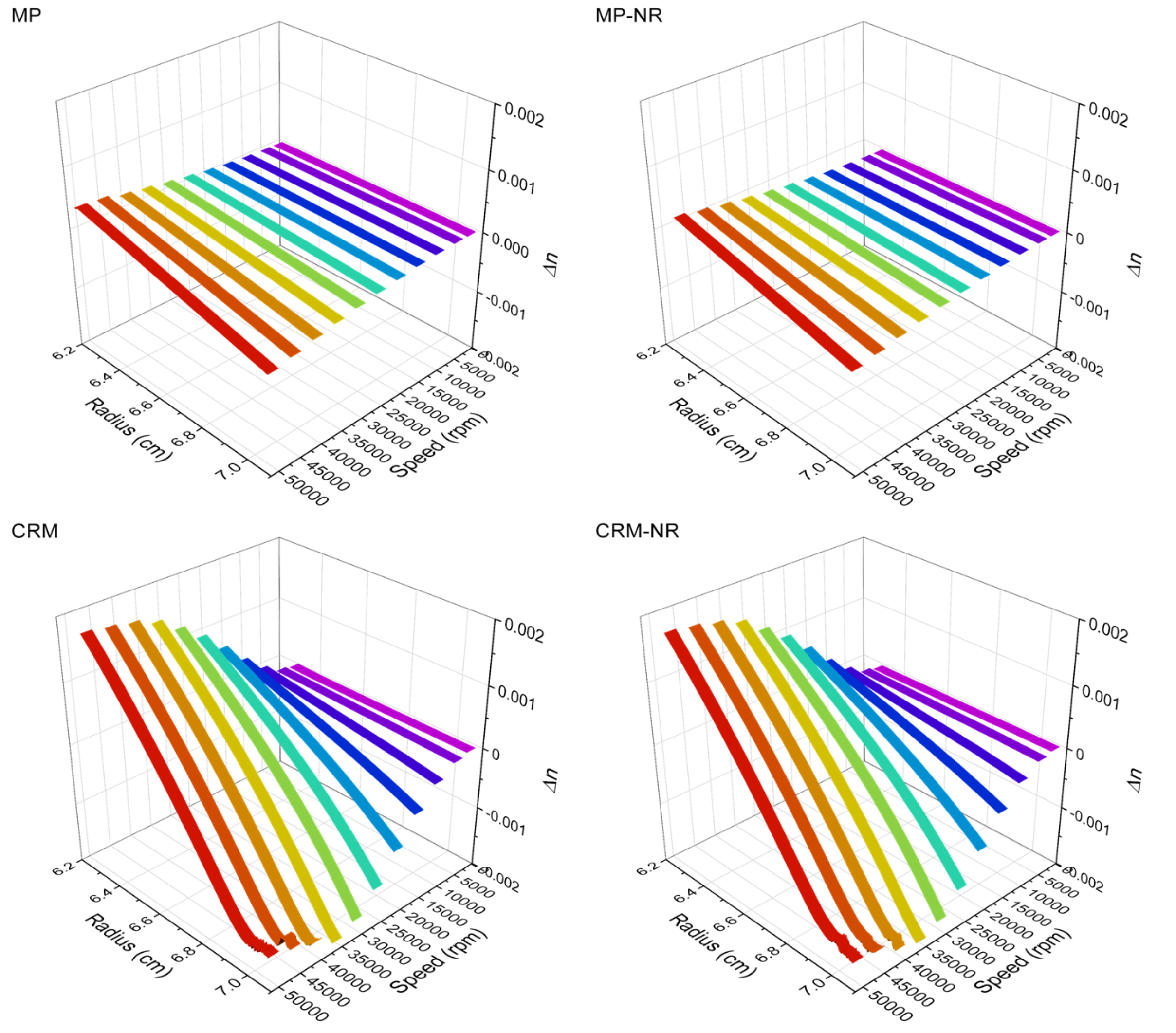

Figure S5. Comparison of a nearly regular solution and a system with pre-ouzo effect: From interference data of flotation equilibria, the recalculated refractive index profile Δn versus radial distance of the measured cell from the rotor centrum (radius, r) and different speeds for the MP experimental point for the ethyl acetate/ethanol/water ternary system and the CRM experimental point for the n-dodecane/ethanol/water ternary system with (right) and without (left) Nile red at 25 °C.

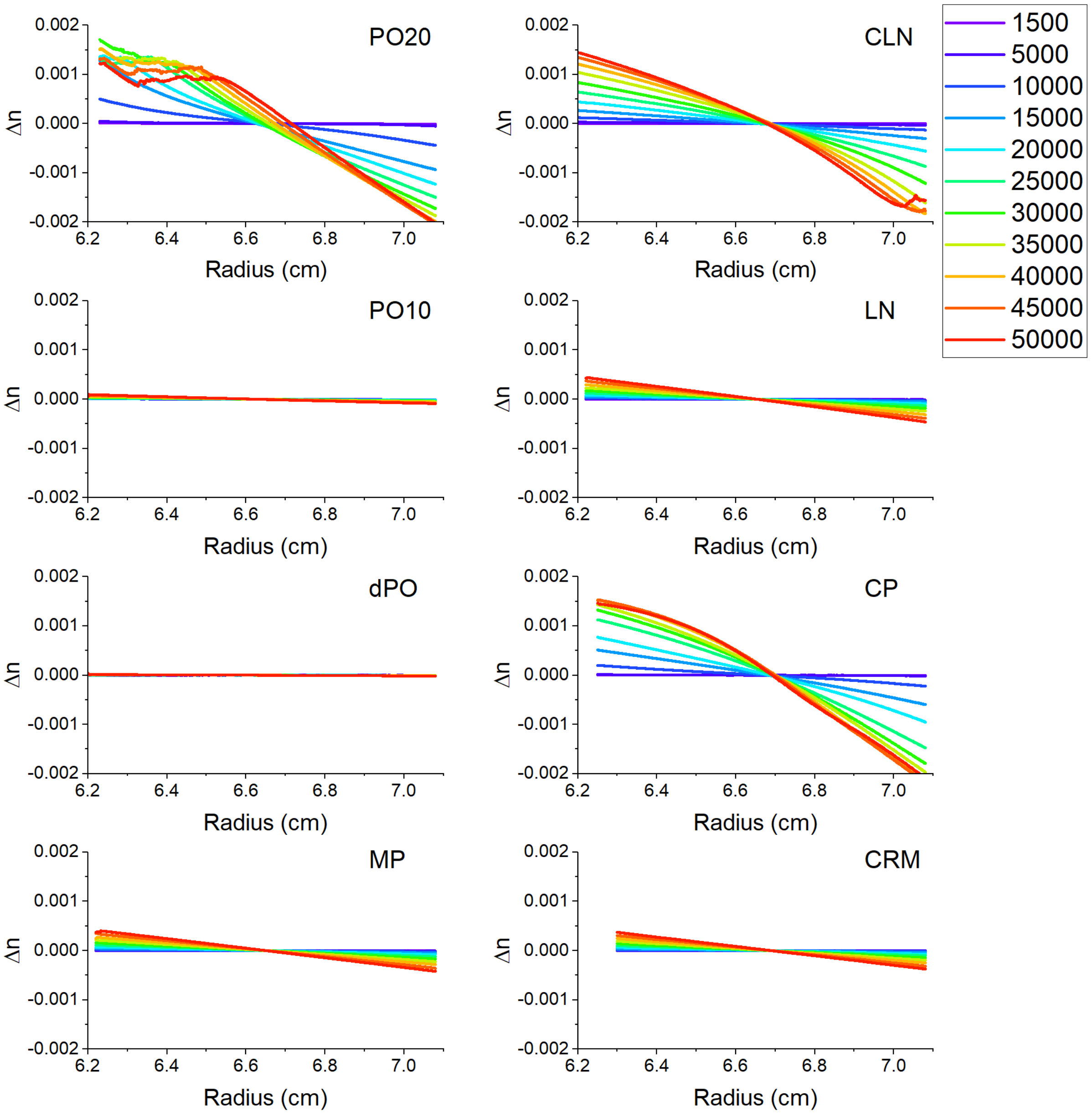

Figure S6. Refractive index profile Δn recalculated from the interference fringe versus radial distance of the measured cell from the rotor centrum (radius, r) as 2D plot.

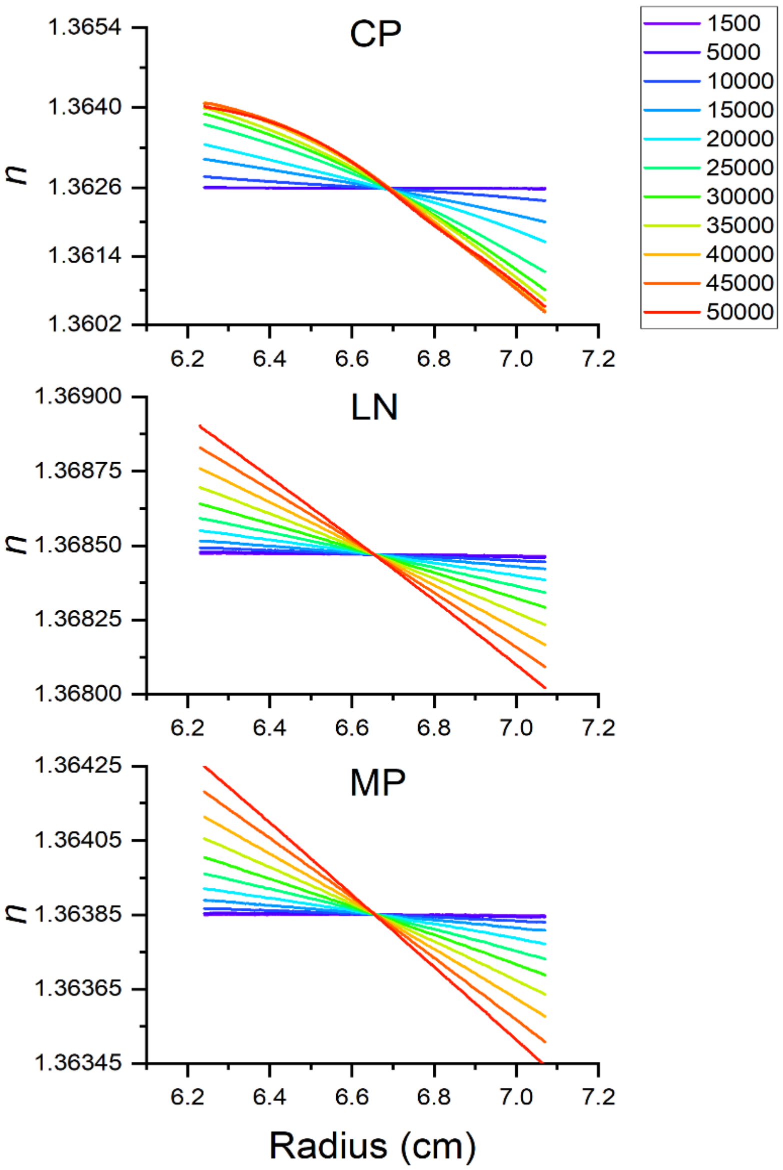

Figure S7. Refractive index n recalculated from the experimental Δn (see Fig. S6) versus radial distance of the measured cell from the rotor centrum (radius, r). The computation of the contributions was done with $n = \Delta n + n_0$, where n_0 is the refractive index of the initial experimental sample composition (also see Figure S9).

Table S1. Molar mass (M_1), densities of sedimenting macromolecule (ρ_1) and solvent mixture (ρ_0), and absolute number of fringes at the bottom of cell (J_a), which are necessary for the calculation of the apparent weight-averaged molar mass ($M_{w,app}/M_1$) for experimental points PO20, CP and CLN at 25 °C.

Exp. Sample	1-Component	M_1 (g/mol)	Densities (g/cm^3)		J_a	$M_{w,app}/M_1$
			ρ_1	ρ_0		
PO20	EA/EtOH	69.36[a]	0.8149[a,b]	0.99705	- 0.64	326.8
CP	Water	18.015	0.99705	0.9079[c]	- 80.24	59.9
CLN	Water	18.015	0.99705	0.8622[b]	- 89.83	47.1

a) This result was calculated with the initial mass composition of the experimental PO20 sample composition (s. Table S4) for binary case of ethyl acetate/ethanol mixture of 0.4348 total mass, which means that 0.554 wt.-% of ethyl acetate and 0.446 wt.-% of ethanol were in initial binary solution

b) result of $\rho_{0(binary)}$ was fitted to a polynomial of the second type and presented in figure S16-B

c) result of $\rho_{0(ternary)}$ was measured with a Density Meter DMA 5000 M (Anton Paar) at 25 °C and also presented in Figure S8

Table S2. Molar mass (M_I), refractive indices (n), densities (ρ), viscosities (η) and relative dielectric constants (ε) for pure liquids at 25 °C.

Component	M_I[a] (g/mol)	n		ρ (g/cm^3)		η (mPa s)	ε
		Exp.[b]	Lit.[a]	Exp.[c]	Lit.[a]	Lit.[a]	Lit.[a]
Water	18.015	1.33251	1.33250	0.99702	0.99705	0.89025	78.36
Ethanol	46.069	1.35937	1.35941	0.78520	0.78493	1.0826	24.55
Ethyl acetate	88.106	1.36981	1.36978	0.89463	0.89455	0.426	6.02

a) Ref. 1

b) result was measured with a Refractometer RX-5000α (Atago) at 25 °C

c) result was measured with a Density Meter DMA 5000 M (Anton Paar) at 25 °C

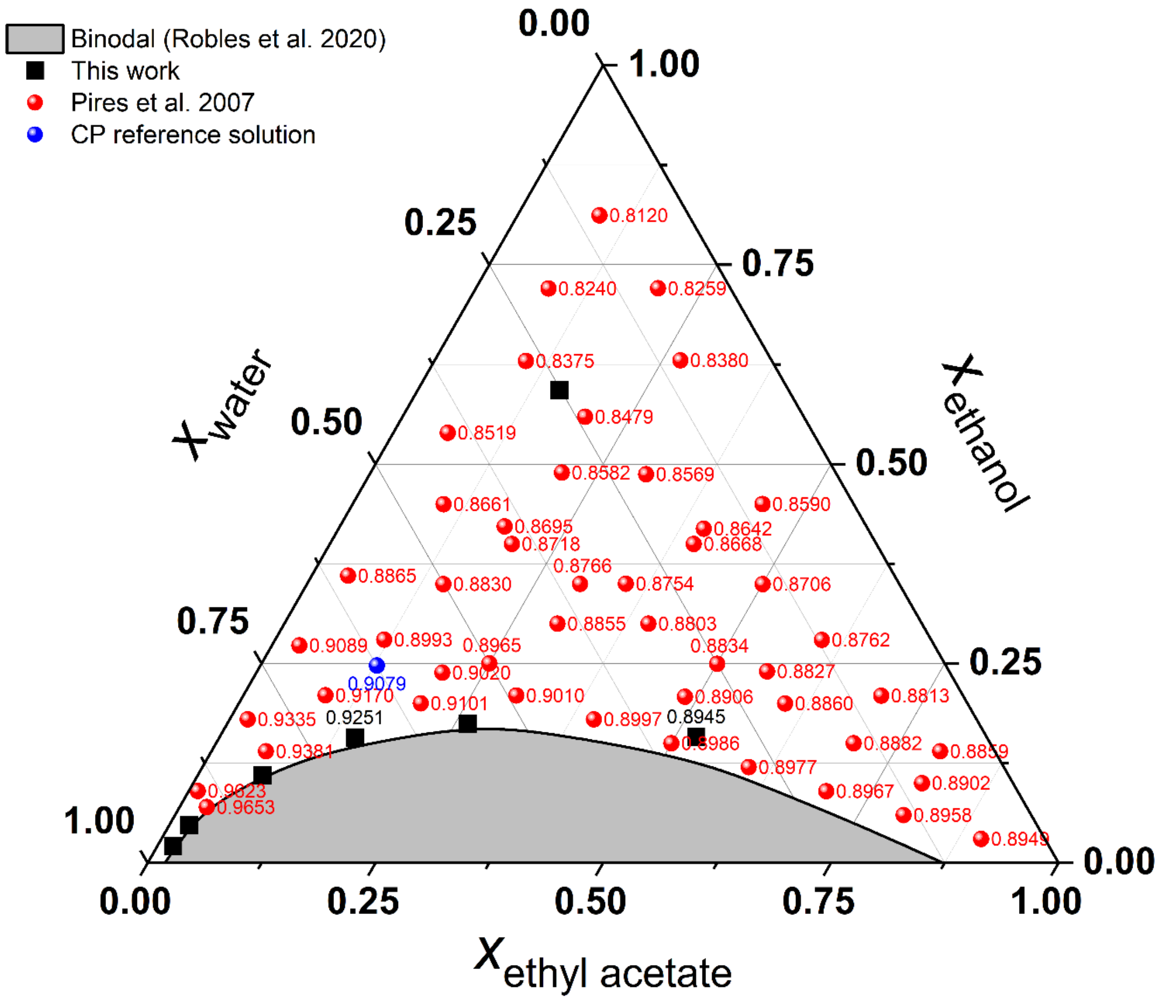

Figure S8. Densities of the ethyl acetate/ethanol/water ternary system at 25 °C obtained by Pires and co-workers[10] (red spheres). The black cubes represent experimental points in this work. The blue sphere represents the measured density value for the reference solution of CP sample.

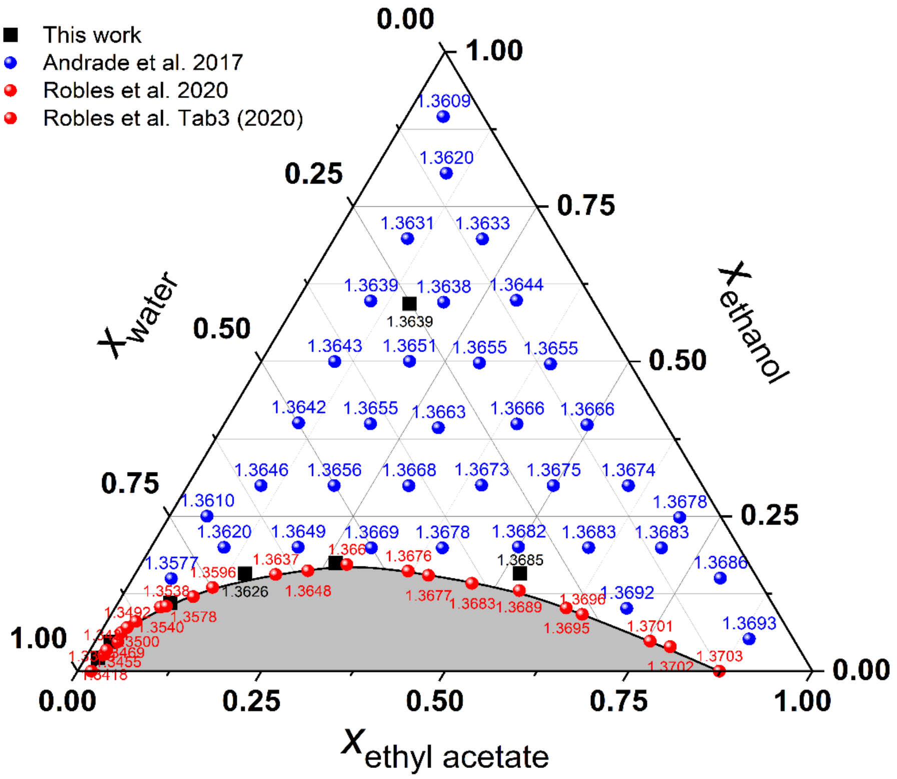

Figure S9. Refractive indices n of the ethyl acetate/ethanol/water ternary system at 25 °C obtained by Andrate and co-workers[7] (blue spheres) and Robles *et al.*[11] (red spheres). The black cubes represent experimental points in this work.

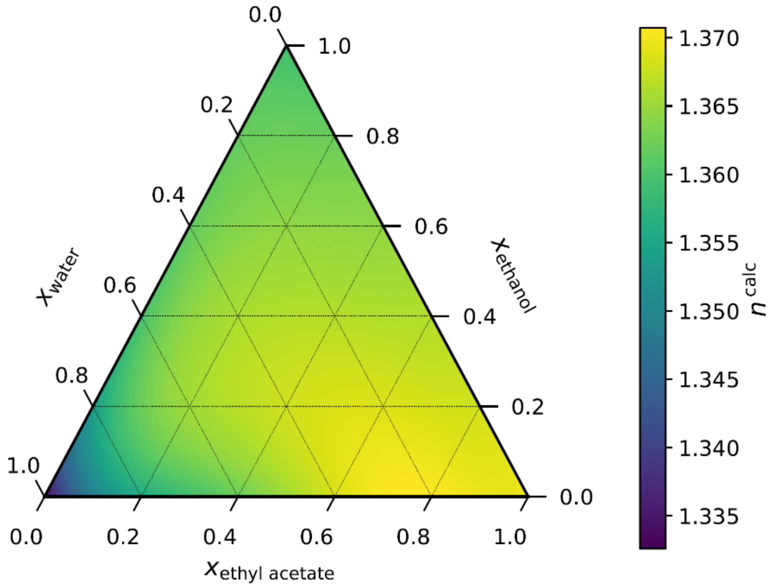

Figure S10. Modelled refractive index n in the ethyl acetate/ethanol/water ternary system at 25 °C.

Used Software

Both, calculations and visualization are done with Python 3.7 and additional libraries. The composition profile is calculated by iteratively calling a root finding algorithm on Eq. 3. Practically, the root algorithm of the scipy.optimize package[2] was used, which applies a modified Powell method[3]. The initial composition was then optimized to yield the desired overall sample composition using the minimize algorithm from the scipy.optimize package. It uses the quasi-Newton method of Broyden, Fletcher, Goldfarb, and Shanno (BFGS)[4]. For plotting, the libraries matplotlib[5] and ternary-python[6] are used.

Refractive index

The calculation follows Andrade et al.[7] The experimentally measured refractive index n deviates from an ideal one, where the ideal one is calculated as

$$n_{\text{ideal}} = \sum_{i=1}^{N} x_i \, n_i \tag{S1}$$

and thus, the deviation as

$$\delta n = n - \sum_{i=1}^{N} x_i \, n_i \tag{S2}$$

Following the naming convention of the literature, the deviation of the refractive index is denoted δQ. To model it in the binary case with the components i and j, a Redlich-Kister (RK) type equation is used:

$$\delta Q_{ij} = x_i x_j \sum_{p=0}^{m} B_p \left(x_i - x_j\right)^p \tag{S3}$$

where the limit of the expansion m is set to 3 and B_p are the fitting parameters. With all the binary expressions known, the ternary deviation can be fitted using

$$\delta \mathrm{Q}_{ijk} = \delta \mathrm{Q}_{ij} + \delta \mathrm{Q}_{ik} + \delta \mathrm{Q}_{jk} + \mathrm{x}_i \mathrm{x}_j \mathrm{x}_k \left(\mathrm{C}_1 + \mathrm{C}_2 \cdot \mathrm{x}_i + \mathrm{C}_3 \cdot \mathrm{x}_j\right) \tag{S4}$$

Due to the missing specification of the fitted parameters in the original literature, own parameters were fitted based on measurements in the binary systems water-ethanol[8] and ethyl

acetate-ethanol[9], and own measurements of the ternary system. After fitting the two sets of binary parameters, the final binary parameters were fitted simultaneously with the ternary parameters using ternary system data. The parameters can be found in Tab. S3. The root mean square deviations was calculated to be 6.1×10^{-5}.

Table S3. Fitting parameters for the binary RK expressions (Eq. S3) and the ternary deviation (Eq S4).

	B_0	B_1	B_2	B_3
ethanol-water	0.05859847	-0.03607098	0.03709852	-0.01143614
ethyl acetate-ethanol	0.00360833	-0.00124314	-0.00053527	0.00021226
ethyl acetate-water	0.05530324	-0.00741702	0.05185450	-0.08249711

		C_1	C_2	C_3
		-0.10076252	0.06029355	0.25263675

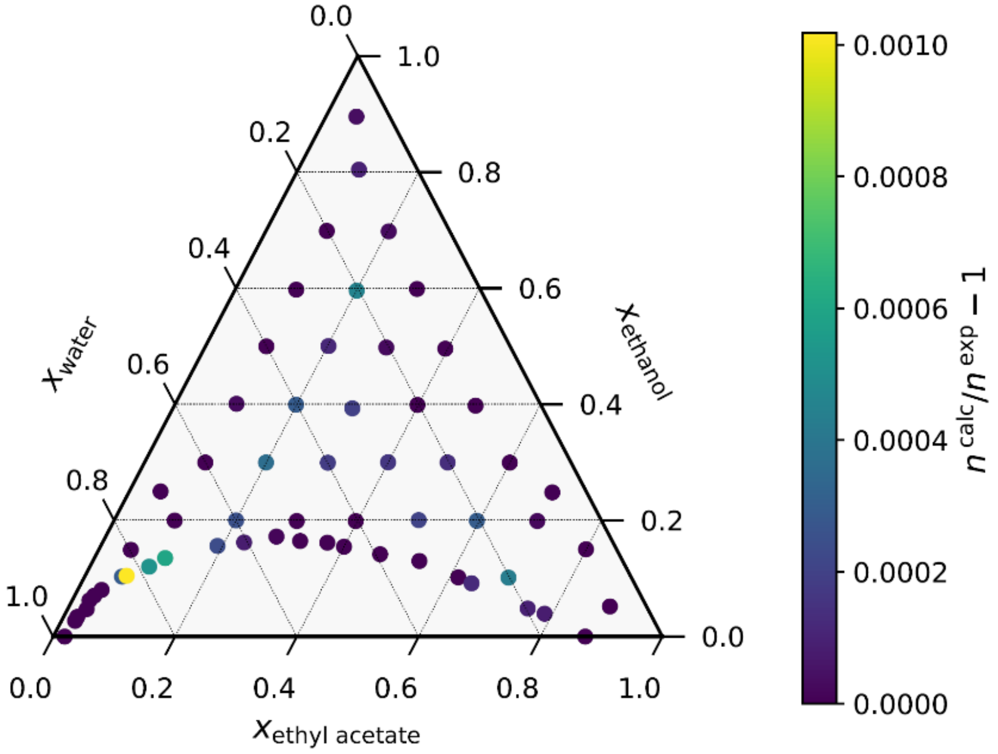

Figure S11. Relative error of calculated refractive index at 25 °C compared to the combined experimental values of Andrade *et al.*[7] and Robles *et al.*[11]

Table S4. Experimental compositions (mass, *w* and mole, *x* fraction) of the ethyl acetate (EA)/ethanol (EtOH)/water (H_2O) mixtures und there reference solutions at 25 °C and atmospheric pressure.

Sample	*w*			*x*		
	EA	EtOH	H_2O	EA	EtOH	H_2O
Without Nile Red						
dPO	0.0764	0.0490	0.8746	0.0172	0.0211	0.9617
PO10	0.0913	0.1040	0.8047	0.0216	0.0471	0.9313
PO20	0.2408	0.1940	0.5652	0.0713	0.1099	0.8187
CP	0.3993	0.2206	0.3801	0.1490	0.1574	0.6936
CLN	0.5613	0.1945	0.2442	0.2639	0.1749	0.5613
LN	0.7795	0.1233	0.0972	0.5229	0.1583	0.3188
MP	0.3003	0.6001	0.0996	0.1552	0.5931	0.2517
With Nile Red						
dPO-NR	0.0751	0.0475	0.8776	0.0168	0.0204	0.9628
PO10-NR	0.0896	0.1105	0.7999	0.0213	0.0502	0.9286
PO20-NR	0.2426	0.1924	0.5650	0.0719	0.1091	0.8190
CP-NR	0.3996	0.2200	0.3805	0.1491	0.1570	0.6940
CLN-NR	0.5678	0.1907	0.2424	0.2680	0.1724	0.5596
LN-NR	0.7828	0.1181	0.0976	0.5272	0.1518	0.3209
MP-NR	0.2998	0.6005	0.0997	0.1549	0.5933	0.2519
Reference solutions for the AUC measurements						
dPO	-	0.0513	0.9487	-	0.0207	0.9793
PO10	-	0.1018	0.8982	-	0.0424	0.9576
PO20	0.9006	0.0994	-	0.8257	0.1743	-
CP	0.3333	0.3332	0.3335	0.1281	0.2450	0.6269
CLN	0.7501	0.2506	-	0.6102	0.3898	-
LN	0.9006	0.0994	-	0.8258	0.1742	-
MP	0.3986	0.6014	-	0.2574	0.7426	-

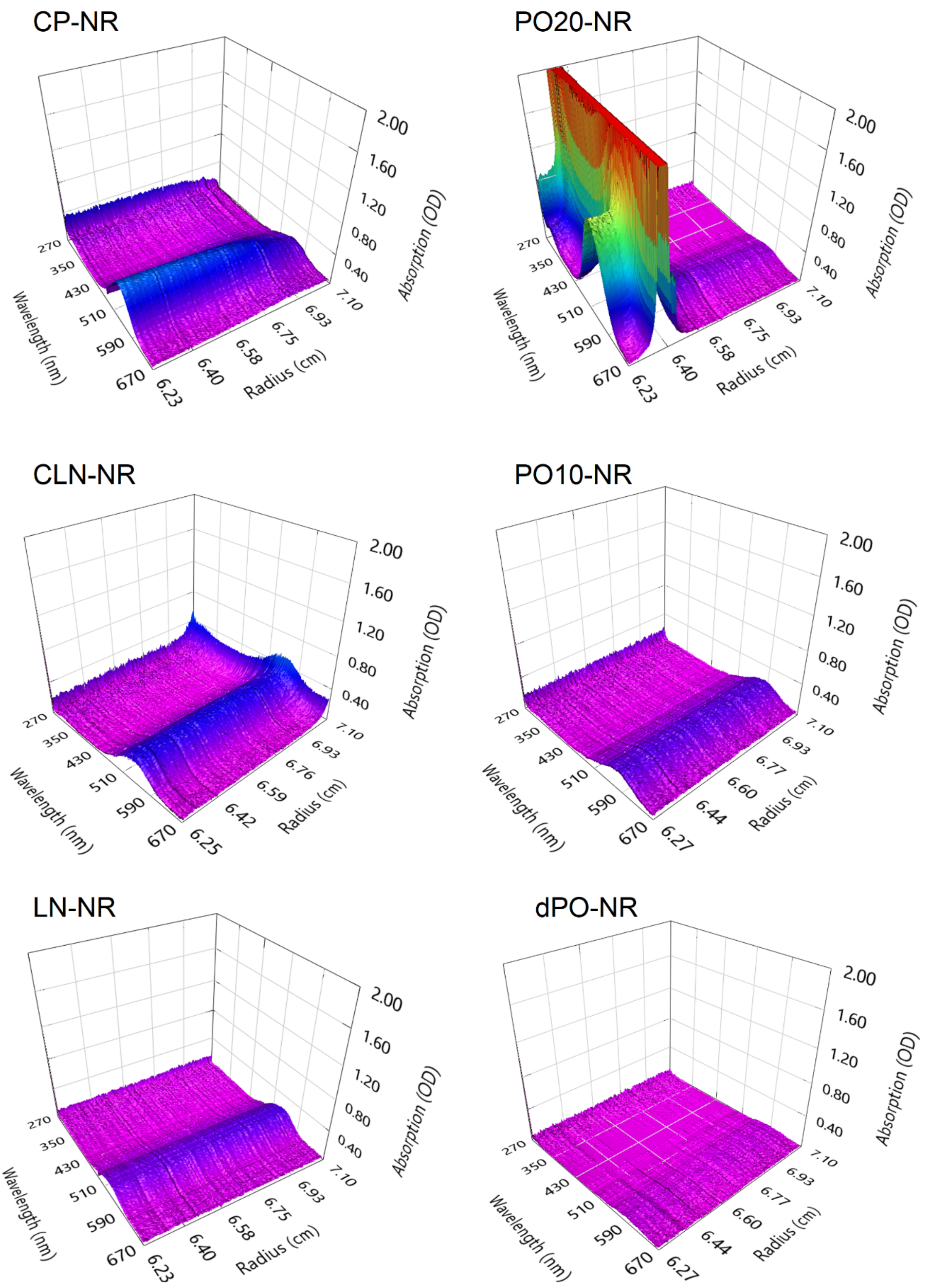

Figure S12. MWL-UV/Vis raw data of sedimentation equilibria at 50 000 rpm and 25 °C for the experimental points for the ternary system ***with*** the presence of Nile red (0.0261 mM ethanol-dye stock solution).

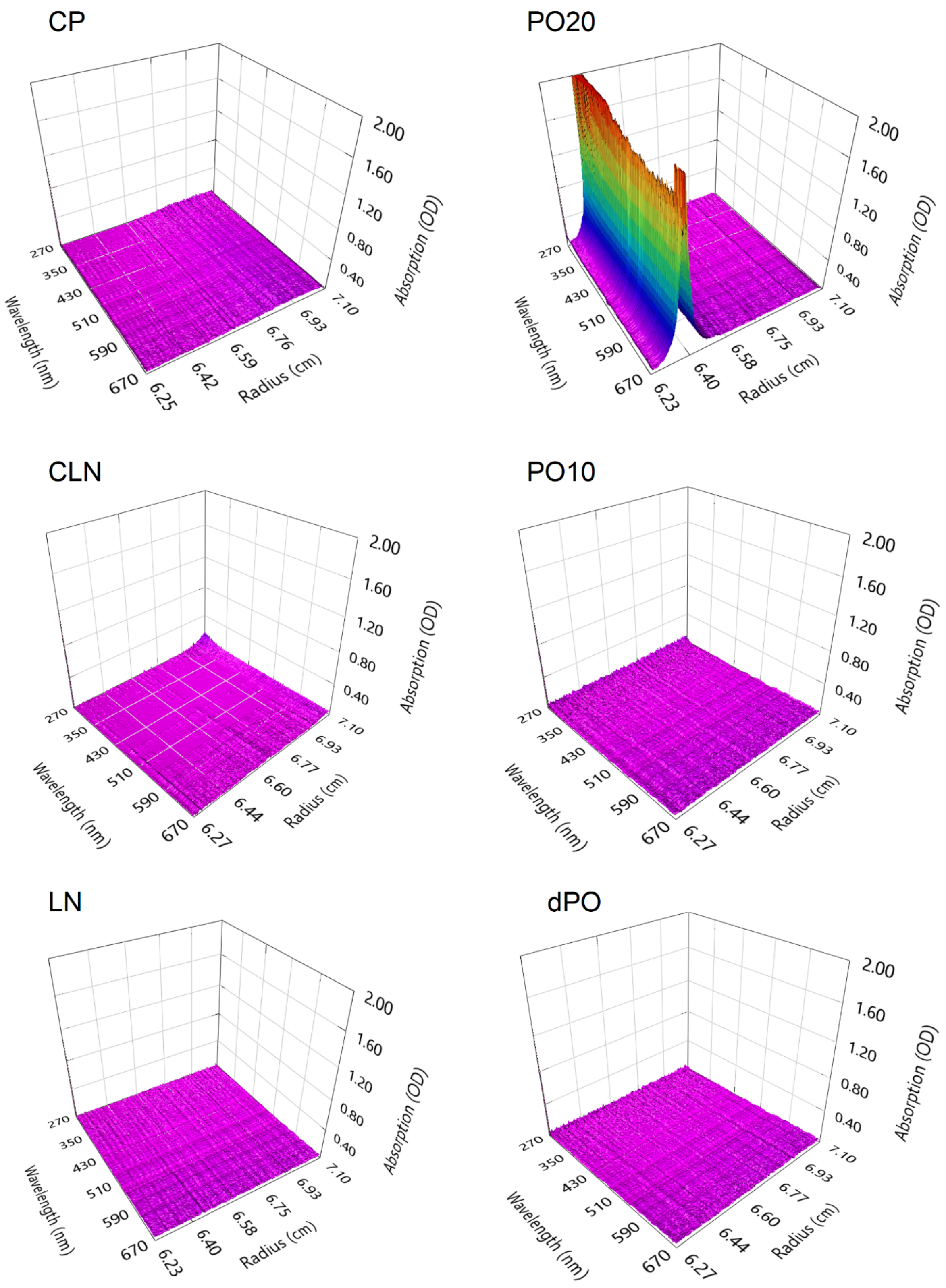

Figure S13. MWL-UV/Vis raw data of sedimentation equilibria at 50 000 rpm and 25 °C for the experimental points for the ternary system ***without*** the presence of Nile red.

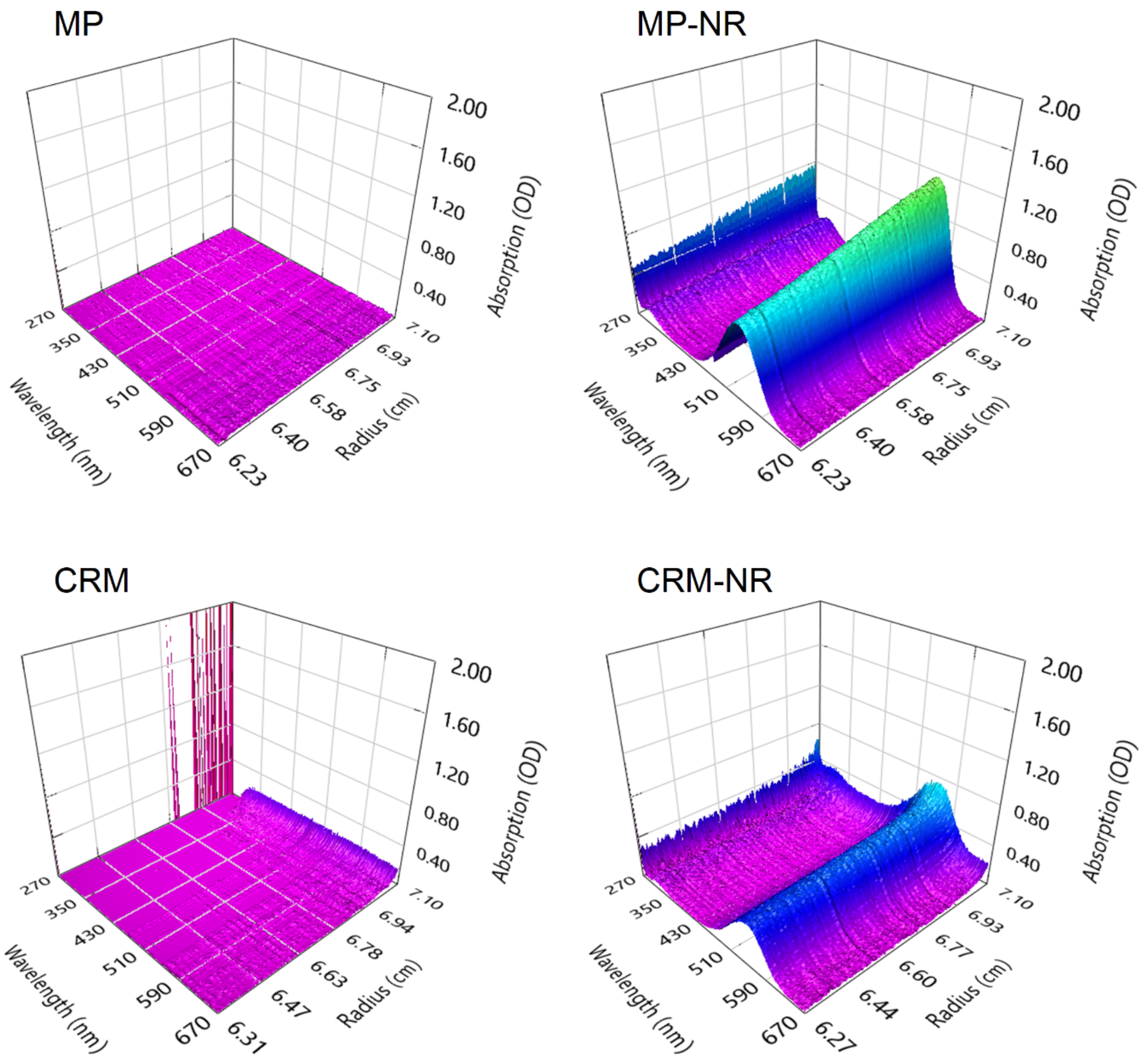

Figure S14. MWL-UV/Vis raw data of sedimentation equilibria at 50 000 rpm and 25 °C for the reference experimental point MP for the ethyl acetate/ethanol/water ternary system and CRM for the *n*-octanol/ethanol/water ternary system with and without the presence of Nile red.

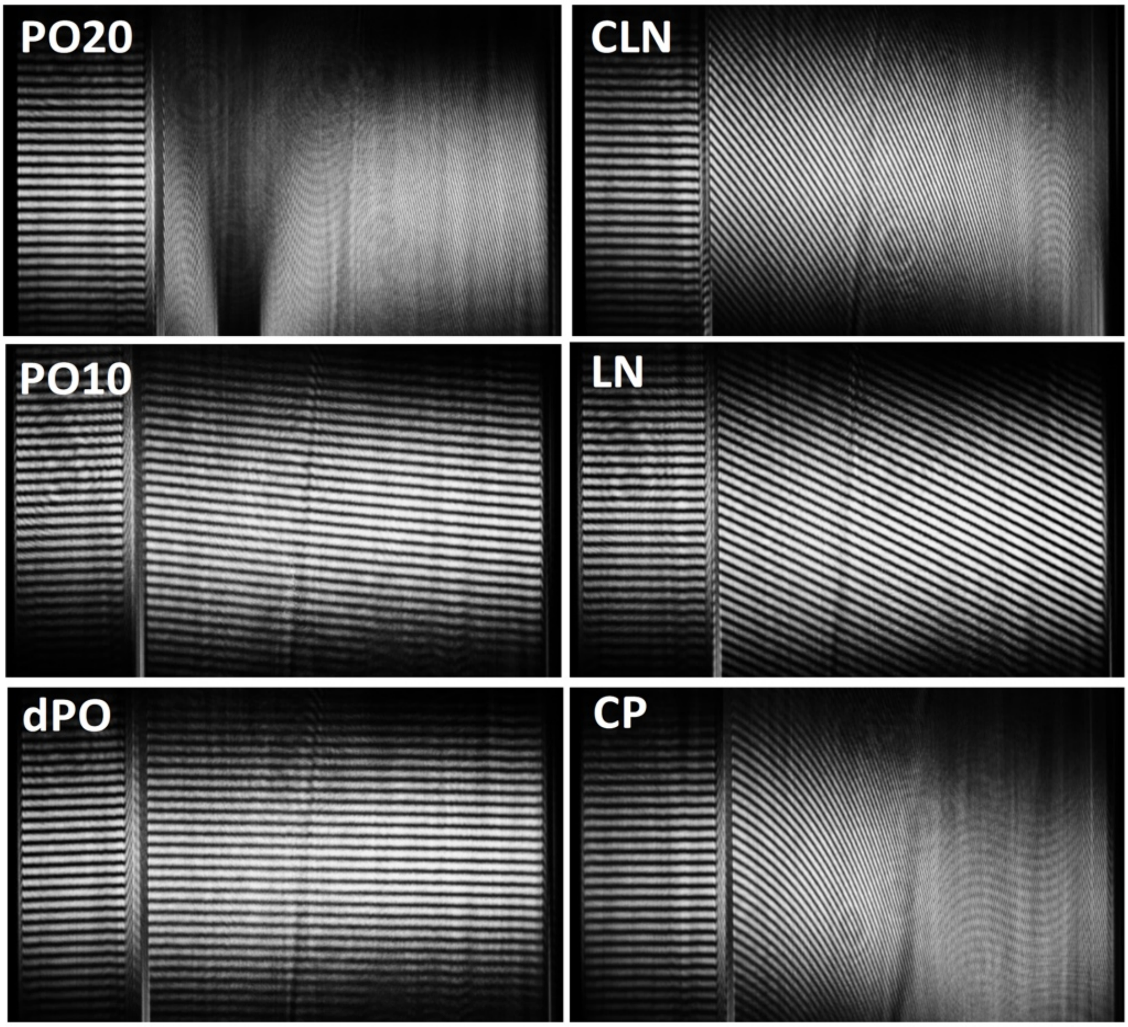

Figure S15. Raw images of the experimentally obtained interference fringe pattern for the ethyl acetate/ethanol/water ternary system at 50 000 rpm and 25 °C.

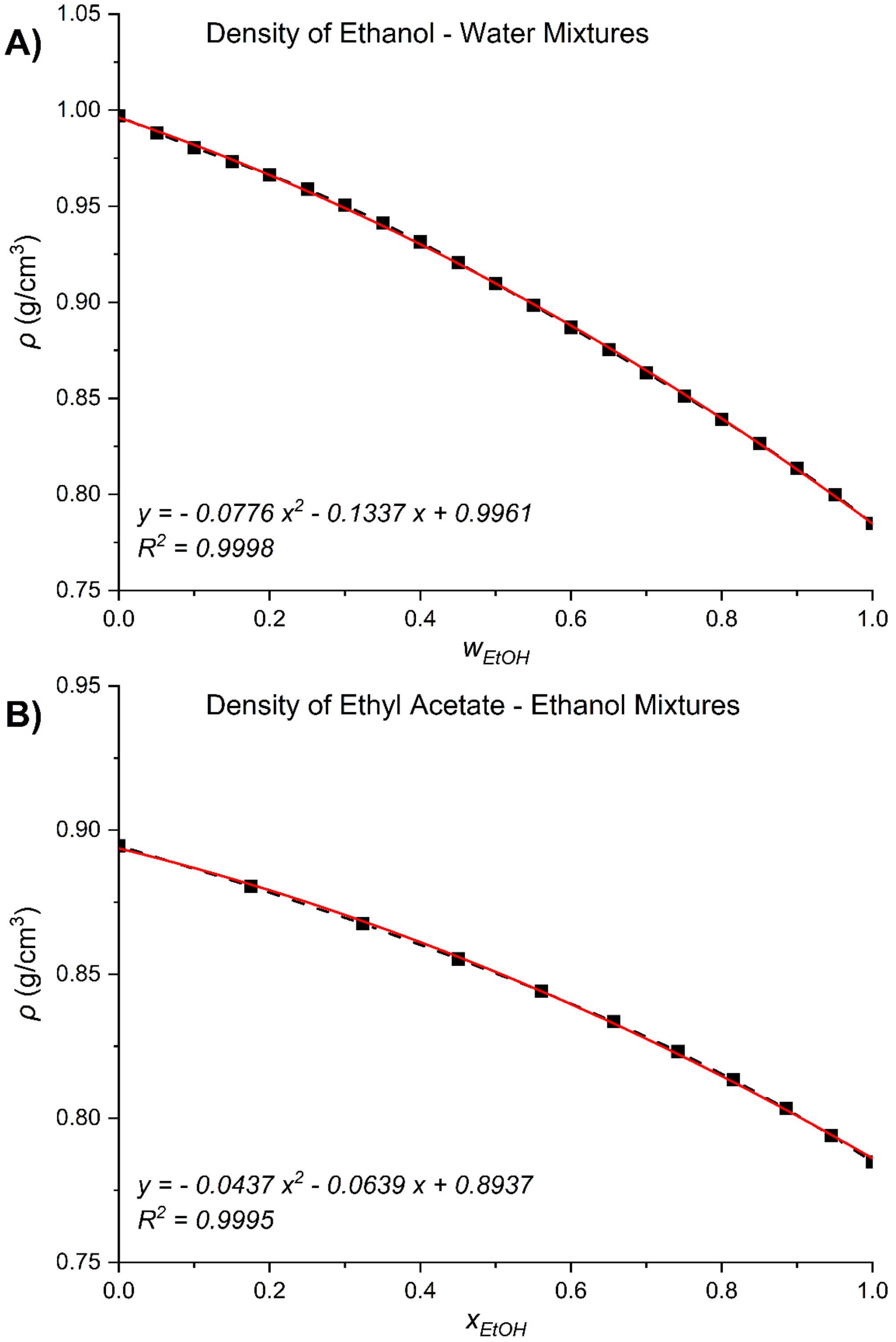

Figure S16. Polynomials of the second type for extrapolating the density of the binary solutions of (A) ethanol/water[12] and (B) ethyl acetate/ethanol[13] at 25 °C.

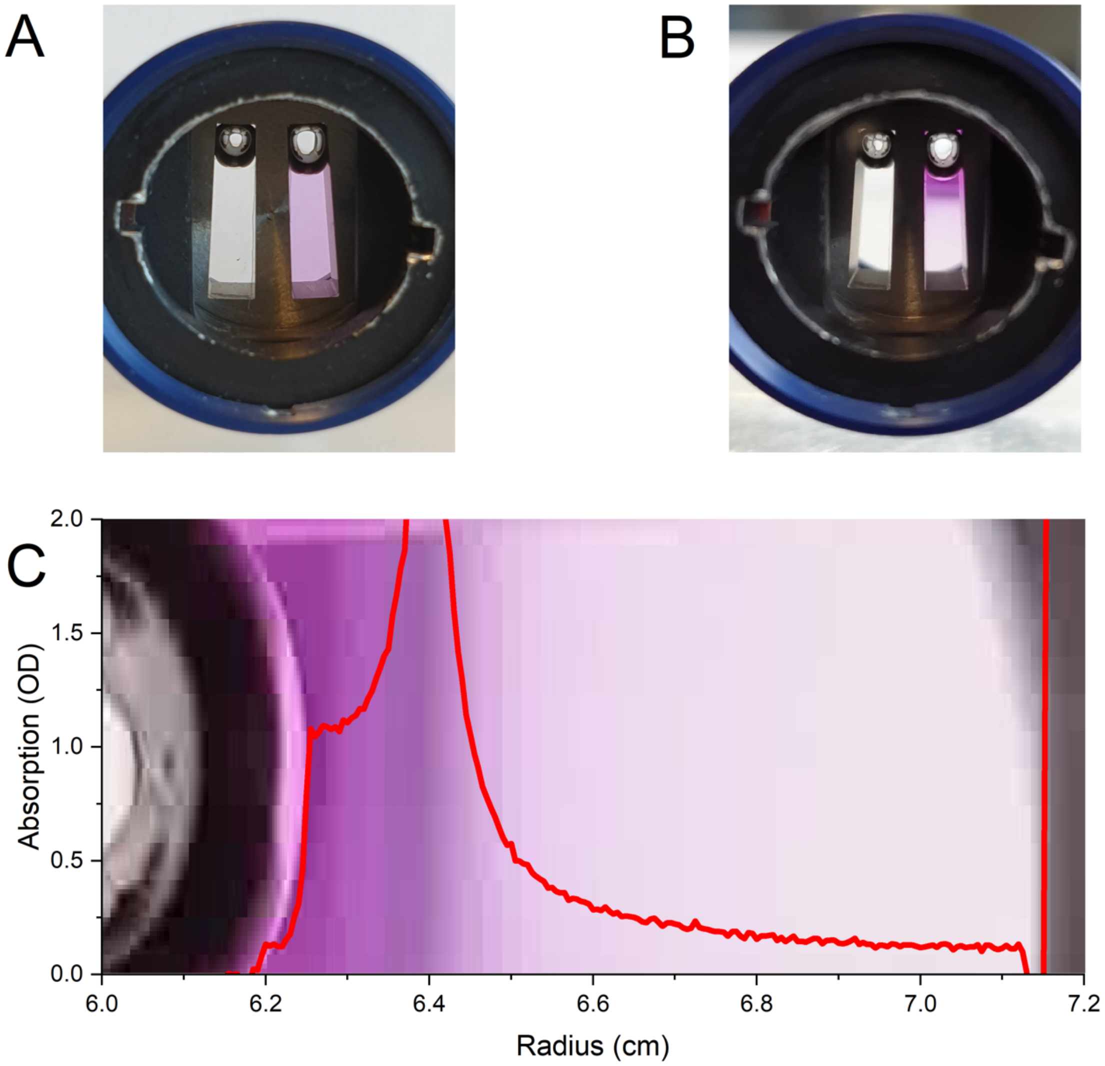

Figure S17. Images of the PO20-NR sample before and after centrifugation at 25 °C in the analytical ultracentrifuge at 50 000 rpm for 94 h. A: 12-mm analytical double-sector cell before centrifugation (left: reference sector with solvent, right: sample sector with PO20 solution with Nile Red). B: Cell after centrifugation with strong concentration gradients in the sample. C: sample sector of B with overlay of measured concentration profile using MWL-UV/Vis absorption optic at 560 nm.

Experimental Uncertainties

For the MWL-UV/Vis optic, the used step motor has a radial step size of 1 µm and a precision of 0.1 µm.

For the interference optic, the accuracy of the Rayleigh interferometer is 0.2 fringes for the optics in air and 0.44 in the presence of the cell. According to

$$\Delta n(r) = \frac{J(r) * \lambda}{a} = 5.625 \times 10^{-5} * J(r)$$

this corresponds to an error of the refractive index of 1.12 x 10^{-5} (Air) and 2.47 x 10^{-5} (with cell).

References:

(1) Riddick, J. A.; Sakano, T.; Bunger William B.; Weissberger Arnold. *Organic solvents : Physical Properties and Methods of Purification*, 4th ed.; Wiley New York, 1986.

(2) Virtanen, P.; Gommers, R.; Oliphant, T. E.; Haberland, M.; Reddy, T.; Cournapeau, D.; Burovski, E.; Peterson, P.; Weckesser, W.; Bright, J.; van der Walt, S. J.; Brett, M.; Wilson, J.; Jarrod Millman, K.; Mayorov, N.; Nelson, A. R. ~J.; Jones, E.; Kern, R.; Larson, E.; Carey, C. J.; Polat, \.Ilhan; Feng, Y.; Moore, E. W.; Vand erPlas, J.; Laxalde, D.; Perktold, J.; Cimrman, R.; Henriksen, I.; Quintero, E. ~A.; Harris, C. R.; Archibald, A. M.; Ribeiro, A. H.; Pedregosa, F.; van Mulbregt, P.; Contributors, S. 1. 0. SciPy 1.0: Fundamental Algorithms for Scientific Computing in Python. *Nat. Methods* **2020**, *17*, 261–272. https://doi.org/https://doi.org/10.1038/s41592-019-0686-2.

(3) Moré, J.; Garbow, B.; Hillstrom, K. User guide for MINPACK-1. 1980.

(4) Nocedal, J.; Wright, S. J. *Numerical Optimization: Springer Series in Operations Research and Financial Engineering*; Springer, 2006.

(5) Hunter, J. D. Matplotlib: A 2D graphics environment. *Comput. Sci. Eng.* **2007**, *9* (3), 90–95. https://doi.org/10.1109/MCSE.2007.55.

(6) Harper, M.; Weinstein, B.; Simon, C.; Chebee7i, S.-H.; Badger, T. G.; Greco, M. python-ternary: Ternary Plots in Python. *Zenodo* **2015**, *12*, 17. https://doi.org/10.5281/zenodo.594435.

(7) Andrade, R. S.; Gonzalez, C.; Iglesias, M. Changes of refractive indices for ethanol + water + (ethyl acetate or 1-pentanol) at 298.15 K. *Int. J. Thermodyn.* **2017**, *20* (3), 174–181. https://doi.org/10.5541/ijot.5000310389.

(8) Wohlfarth, C. *Refractive Indices of Pure Liquids and Binary Liquid Mixtures (Supplement to III/38)*; Lechner, M. D., Hrsg.; Landolt-Börnstein - Group III Condensed Matter; Springer Berlin Heidelberg: Berlin, Heidelberg, 2008; Bd. 47. https://doi.org/10.1007/978-3-540-75291-2.

(9) Andreatta, A. E.; Arce, A.; Rodil, E.; Soto, A. Physical properties of binary and ternary mixtures of ethyl acetate, ethanol, and 1-octyl-3-methyl-imidazolium bis(trifluoromethylsulfonyl)imide at 298.15 K. *J. Chem. Eng. Data* **2009**, *54* (3), 1022–1028. https://doi.org/10.1021/je800899w.

(10) Pires, R. M.; Costa, H. F.; Ferreira, A. G. M.; Fonseca, I. M. A. Viscosity and density of water + ethyl acetate + ethanol mixtures at 298.15 and 318.15 K and atmospheric pressure. *J. Chem. Eng. Data* **2007**, *52* (4), 1240–1245. https://doi.org/10.1021/je600565m.

(11) Robles, P. A.; Lourenço, N. I.; Igarashi, E. M. S.; Sousa, M. N.; Arce, P. F. Thermodynamic Behavior of the Phase Equilibrium of Ethyl Acetate + Ethanol + Water Systems at Atmospheric Pressure: Experiment and Modeling. *J. Chem. Eng. Data* **2020**, *65* (4), 1402–1410. https://doi.org/10.1021/acs.jced.9b00785.

(12) *CRC Handbook of Chemistry and Physics: A Ready-Reference of Chemical and Physical Data*, 97th Aufl.; Haynes, W. M., Lide, D. R., Bruno, T. J., Hrsg.; CRC Press: Boca Raton, 2017.

(13) Nikam, P. S.; Mahale, T. R.; Hasan, M. Density and viscosity of binary mixtures of ethyl acetate with methanol, ethanol, propan-1-ol, propan-2-ol, butan-1-ol, 2-methylpropan-1-ol, and 2-methylpropan-2-ol at (298.15, 303.15, and 308.15) K. *J. Chem. Eng. Data* **1996**, *41* (5), 1055–1058. https://doi.org/10.1021/je960090g.
